# The density of Braun’s Lipoprotein determines vesicle production in *E. coli*

**DOI:** 10.1371/journal.pone.0332156

**Published:** 2025-09-19

**Authors:** Brian P. Weaver, Fengjie Zhao, Yuanli Gao, James Q. Boedicker, Christoph A. Haselwandter

**Affiliations:** 1 Department of Physics and Astronomy, University of Southern California, Los Angeles, California, United States of America; 2 Department of Biological Sciences, University of Southern California, Los Angeles, California, United States of America; 3 Department of Quantitative and Computational Biology, University of Southern California, Los Angeles, California, United States of America; University at Buffalo, UNITED STATES OF AMERICA

## Abstract

Production of extracellular vesicles by Gram-negative bacteria is known to be associated with blebbing of the outer membrane. Several proteins within Gram-negative bacteria crosslink the outer membrane to the cell wall, and thereby stabilize the cell envelope. Prior work in *Escherichia coli* demonstrated that crosslinking proteins reduce membrane blebbing and vesicle formation, and that deletion of crosslinking proteins, most notably Braun’s lipoprotein (Lpp), can increase vesicle production by about two orders of magnitude. To examine the quantitative relation between crosslinking proteins and bacterial vesicle formation, we develop here a simple physical model that predicts how vesicle production depends on the Lpp density. We test our model by measuring vesicle production in a strain of *E. coli* with tunable Lpp expression. Our experimental observations agree with our model predictions for most measured Lpp densities. For low Lpp densities, our experiments yield more pronounced vesicle production than predicted by our model, which can be explained if the mechanical properties of the cell envelope depend on the Lpp density. Our results shed light on basic principles and molecular mechanisms governing bacterial vesicle production.

## Introduction

Bacterial vesicles are small, spherical structures produced by a wide range of bacterial species [1–3]. The production and uptake of bacterial vesicles facilitate the exchange of biomolecules, including molecular signals and genetic material [4–9]. Bacterial vesicles can also serve as decoys to protect bacteria from phage infection and assist in the disposal of misfolded proteins [10,11]. Bacterial vesicle biogenesis can be the result of outer membrane blebbing, although other pathways for bacterial vesicle formation have also been reported, including explosive cell lysis [12,13]. There are no known molecular mechanisms that solely regulate bacterial vesicle production, but many genes and pathways are known to impact the propensity for vesicle formation. In particular, the molecular composition and organization of the bacterial cell envelope influence bacterial vesicle production. For example, bacterial vesicle production increases in response to deletion of genes that control the lipid composition of the outer membrane [14,15]. Furthermore, bacterial vesicle production increases in the presence of biomolecules that bind to and restructure cell membranes [16–18].

Intriguingly, bacterial vesicle production strongly increases upon deletion of genes that control the production of proteins that crosslink the outer membrane to the cell wall [19]. Notably, Braun’s lipoprotein (Lpp), which connects the cell wall to the outer membrane in *Escherichia coli*, strongly affects bacterial vesicle production. Lpp is anchored within the outer membrane and covalently attaches to diaminopimelic acid in the bacterial cell wall [20], a process mediated by a family of L,D-Transpeptidases [21]. Lpp is one of the most prevalent proteins in *E. coli*, with individual cells expressing between 500,000 and 750,000 copies [22]. Lpp helps to maintain the rod shape of the *E. coli* outer membrane, and plays an important role in ensuring envelope integrity [23]. Deletion of Lpp results in an approximately 150-fold increase in vesicle production in *E. coli*, the largest increase in vesicle production reported for any single gene deletion in *E. coli* [14]. *E. coli* regulate the number of Lpp via a regulatory mRNA, MicL, which prevents translation of *lpp* mRNA in response to Lpp overproduction, and control the level of crosslinking via a cleaving enzyme, LdtF, which is also called DpaA [24–26].

We employ here a combination of physical modeling and in vivo experiments to quantify the relation between Lpp crosslinks and vesicle formation in *E. coli*. Previous studies have proposed that bacterial vesicle formation occurs in outer membrane regions lacking crosslinking proteins [13,19,27,28]. We refer here to such outer membrane regions as ‘untethered membrane regions.’ We first provide quantitative estimates of the size distributions of untethered membrane regions in *E. coli*. We then develop a simple model of bacterial vesicle formation built on the assumption that vesicles form primarily through large untethered membrane regions. Combining this model with our estimates of the size distributions of untethered membrane regions, we predict how vesicle production in *E. coli* changes with the Lpp density. To test our model predictions, we designed an experimental system that allows us to systematically reduce the Lpp density in *E. coli*. We find that our model successfully predicts the observed fold changes in vesicle production at most induced expression levels of Lpp. For low Lpp densities, our experiments yield a larger-than-predicted increase in vesicle production. We suggest that this discrepancy can be explained if the mechanical properties of the cell envelope depend on the Lpp density.

## Results

### Size distributions of untethered outer membrane regions

**Fig 1:**
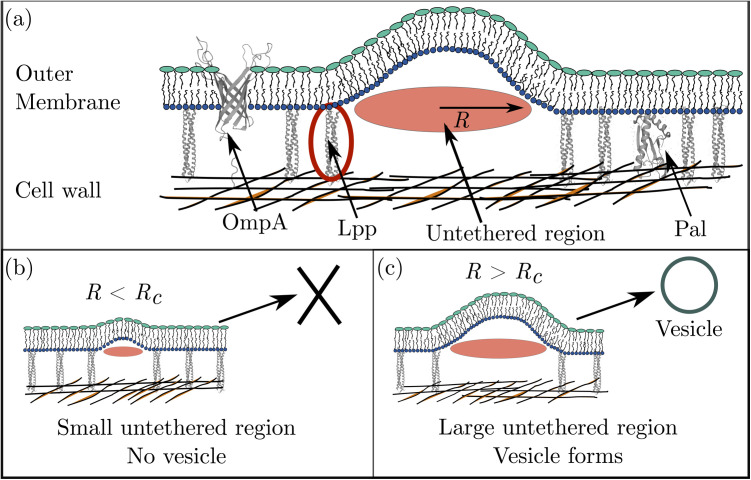
Schematic of bacterial vesicle production. (a) The cell envelope of Gram-negative bacteria consists of the outer membrane, cell wall, and inner (cytoplasmic) membrane (not shown). The outer membrane is attached to the cell wall by crosslinking proteins such as Lpp, OmpA, and Pal. Of these crosslinking proteins, Lpp is the most prevalent in *E. coli*. Gaps between protein crosslinks create untethered membrane regions, as illustrated by the circle of radius R. (b) We assume here that vesicles have a low propensity to form through small untethered membrane regions, $R<R_{c}$. (c) Conversely, we assume that vesicles have a relatively high propensity to form when there is sufficient space between crosslinks, $R>R_{c}$. The Lpp, OmpA, and Pal protein structures illustrated here are based on Refs. [29–31].

Our model of bacterial vesicle production is rooted in the notion that membrane bulging is locally inhibited by the presence of crosslinks tethering the outer membrane to the cell wall [see Fig 1(a)]. In particular, we expect that the large energy cost of breaking covalent bonds linking Lpp to the cell wall makes vesicle formation highly unfavorable in membrane regions tethered to the cell wall. In keeping with refs [13,19,27,28] we therefore assume that vesicle formation occurs primarily in untethered membrane regions. Furthermore, we reason that membrane bulging will be unfavorable in small untethered membrane regions [see Fig 1(b)], where only small deviations from a planar shape are expected to occur spontaneously, with vesicle formation occurring primarily in large untethered membrane regions [see Fig 1(c)]. Indeed, as mentioned above, substantially more vesicles are formed if Lpp is deleted [14], which is expected to decrease the number of untethered membrane regions but increase their size.

The assumption that, for vesicles to form, untethered membrane regions need to be ‘large enough’ can be quantified by assuming that vesicle formation occurs primarily in untethered membrane regions with radii R greater than some critical radius $R_{c}$ [Fig 1(c)]. In this section we use available data on proteins crosslinking the *E. coli* outer membrane and cell wall to provide quantitative estimates of the R-distributions of untethered membrane regions in *E. coli*. In the following sections, we then use these R-distributions to predict, as a function of the density of crosslinking proteins, the fold change in bacterial vesicle production with respect to wild-type (WT) *E. coli*, and compare these predictions to experiments quantifying vesicle production in *E. coli*.

*E. coli*, like many Gram-negative bacteria, have multiple proteins that crosslink the cell wall to the outer membrane. Notably, in addition to Lpp, outer membrane protein A (OmpA) and the peptidoglycan associated lipoprotein (Pal) are known to connect the *E. coli* outer membrane to the cell wall. Roughly, there can be up to 750,000 Lpp [19], 100,000 OmpA [32], and 20,000 Pal [33] proteins in a single cell of *E. coli*. At first glance, these approximate upper bounds on the number of crosslinking proteins suggest that there is an enormous number of crosslinks connecting the *E. coli* outer membrane to the cell wall. However, a single crosslink is often composed of multiple crosslinking proteins. For Lpp, functional crosslinks were found to be trimers [34], and two thirds of the Lpp expressed in *E. coli* are thought to exist in a transmembrane conformation that is not attached to the cell wall [35]. Molecular simulations have indicated that OmpA must dimerize to form a crosslink, and that at least some of these dimers bind to Lpp to form a composite crosslink [36]. Pal, uniquely, is able to crosslink without forming a complex, but even so shows some degree of interaction with Lpp and OmpA, as well as other members of the Tol-Pal complex, such as TolB [33,37]. We thus estimate that there may be as many as 110,000 crosslinks in a given WT *E. coli* cell, with an average spacing of roughly 7 nm [see the Methods section]. Up to about 84,000 of these crosslinks are associated with Lpp. For the computations described here, we used these upper bounds on the number of crosslinking proteins. We find that, if fewer crosslinks are present in WT cells, our model yields similar results for the dependence of bacterial vesicle production on Lpp density, provided that $R_{c}$ is shifted to larger values [see, for instance, S1 Fig]. Thus, the specific numerical values of $R_{c}$ considered here should be viewed as lower bounds on $R_{c}$.

**Fig 2:**
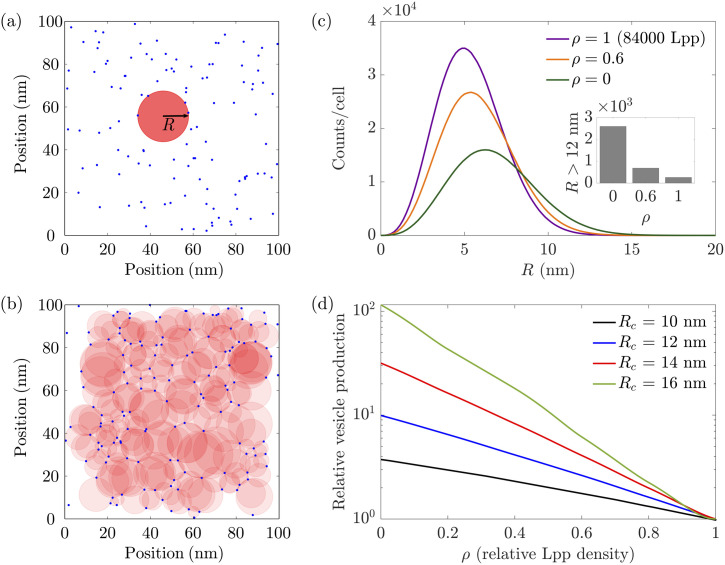
The crosslink density sets the size of untethered membrane regions and, hence, determines vesicle production in our model. (a) Example of an untethered, circular membrane region of radius R. (b) Illustration of a crosslink distribution in which all untethered membrane regions were identified using a Voronoi tessellation. (c) R-distribution of untethered membrane regions, N(R,ρ), for three different Lpp densities ρ, assuming a random crosslink distribution and an *E. coli* outer membrane area equal to $\text{6 }\mu {\text{m}}^{\text{2}}$. (inset) Estimates of the number of untethered membrane regions per *E. coli* cell with $R>R_{c}=\text{12 nm}$ for ρ=0, 0.6, and 1. (d) Predicted fold change in bacterial vesicle number with respect to WT E. coli, f(ρ) in Eq. (1), as a function of Lpp density for $R_{c}=\text{10 nm}$, $R_{c}=\text{12 nm}$, $R_{c}=\text{14 nm}$, and $R_{c}=\text{16 nm}$.

To model the distribution of crosslinks in *E. coli* we randomly place crosslinks associated with Lpp, Pal, and OmpA over a two-dimensional surface representing the outer membrane [see Fig 2(a)]. We keep the density of Pal crosslinks fixed irrespective of the Lpp expression level while, to account for the binding of OmpA to Lpp, we assume that the density of functional OmpA crosslinks varies with the Lpp crosslink density [see the Methods section]. We allow for variations in the Lpp density from zero, ρ=0, to the WT level of Lpp, which we define as the reference (dimensionless) Lpp density ρ=1. Based on previous experiments implying that crosslinks are distributed homogenously throughout the *E. coli* outer membrane [38] we focus on scenarios in which there are no correlations between the placement of crosslinks. We note, however, that recent AFM measurements suggest that Lpp crosslinks may form clusters [39]. S2 Fig explores the impact of correlations in the crosslink placement on our model results.

At the most basic level, each untethered membrane region can be represented by a circle of radius R containing three crosslinks on its circumference and no crosslinks within its interior [Fig 2(a)]. As illustrated in Fig 2(b), we identify such untethered membrane regions from the simulated crosslink locations using a Voronoi tessellation [see the Methods section]. For a given Lpp density ρ, these untethered membrane regions are then used to generate a distribution of the radii of untethered membrane regions [see Fig 2(c)]. We denote this R-distribution by N(R,ρ). To compute the N(R,ρ associated with a particular ρ, we randomly placed crosslinks over a membrane area of size 100 nm× 100 nm with a crosslink density chosen so as to account for Lpp, Pal, and OmpA crosslinks, and averaged over 100,000 realizations. Repeating this procedure for different Lpp densities we find the characteristic R-distributions associated with different Lpp densities [Fig 2(c)]. Note that, at ρ=0, all the crosslinks connecting the outer membrane to the cell wall are due to OmpA and Pal proteins, with about 85 OmpA and 30 Pal crosslinks per 100 nm× 100 nm membrane patch, respectively. In the next section, we use N(R,ρ) to model the fold change in bacterial vesicle number with respect to WT *E. coli* as the Lpp density is modified.

### Predicting vesicle production from the Lpp crosslink density

Due to the prevalence of Lpp crosslinks in WT *E. coli*, N(R,ρ) shows a pronounced dependence on the Lpp density ρ [Fig 2(c)]. Notably, when the Lpp density is reduced, there is a reduction in the number of untethered membrane regions with small R, and an increase in the number of untethered membrane regions with large R. Assuming that bacterial vesicle production results primarily from untethered membrane regions with $R\geq R_{c}$, we expect these changes in N(R,ρ) to yield an increase in vesicle production with respect to WT cells as ρ is reduced from ρ=1 (WT *E. coli*) to ρ=0 (*E. coli* with no Lpp crosslinks). We quantify changes in vesicle production through the fold change in vesicle production with respect to WT *E. coli*,


$$\begin{array}{c} f(\rho )=\frac{\int_{\text{0}}^{\infty }w(R)\;N(R,\rho )\;dR}{\int_{\text{0}}^{\infty }w(R)\;N(R,\text{1})\;dR}\; \end{array}$$ (1)

where the weight w(R) denotes the propensity for a given untethered membrane region of radius R to form a vesicle. Assuming that vesicles result primarily from untethered membrane regions with large R, w(R) must suppress contributions to the integrals in Eq. (1) due to small R. A simple way to achieve this is to assume that w(R) takes the form $w(R)=\theta \left(R-R_{c}\right)$, where θ(x) is the Heaviside step function. Untethered membrane regions with $R<R_{c}$ then have a propensity of 0, and untethered membrane regions with $R\geq R_{c}$ a propensity of 1. In S1 Text and S3 Fig we explore other functional forms of w(R)—in particular, linear, sigmoidal, and exponential w(R). We find that our model predictions are robust with respect to the precise functional form of w(R), provided that the influence of untethered membrane regions with small R on f(ρ) in Eq. (1) is suppressed sufficiently strongly. For simplicity, we set here $w(R)=\theta \left(R-R_{c}\right)$.

Because the distribution of the radii of untethered membrane regions in *E. coli* depends strongly on the Lpp density [Fig 2(c)], the number of untethered membrane regions with ${R\geq R}_{c}$ and, hence, the fold changes in bacterial vesicle production predicted by Eq. (1) tend to show a pronounced dependence on ρ. To illustrate this point, we show in the inset of Fig 2(c) the predicted number of untethered membrane regions with radii greater than $R_{c}=\text{12 nm}$ for ρ=0, ρ=0.6, and ρ=1. We find that the number of untethered membrane regions with ${R>R}_{c}$ increases by a factor of three as ρ is decreased from ρ=1 to ρ=0.6, and by a factor of ten as ρ is decreased from ρ=1 to ρ=0. Equation (1) then implies that vesicle production at ρ=0.6 and ρ=0 is increased by factors of three and ten with respect to WT *E. coli*, respectively. For a given value of $R_{c}$, our model can thus be used to predict the fold change in bacterial vesicle production as the Lpp density is decreased.

Fig 2(d) explores the predicted fold changes in bacterial vesicle production with respect to WT *E. coli* for Lpp densities ranging from ρ=0 to ρ=1, using $R_{c}=\text{10 nm}$, $R_{c}=\text{12 nm}$, $R_{c}=\text{14 nm}$, and $R_{c}=\text{16 nm}$. Equation (1) is seen to predict that, independent of the specific value of $R_{c}$ considered, vesicle production increases approximately exponentially with decreasing Lpp density. Notably, with $R_{c}\approx \text{16 nm}$, our model predicts that f(ρ) increases by two orders of magnitude as ρ is decreased from ρ=1 to ρ=0. Previous experiments found that deletion of Lpp results in an approximately 150-fold increase in vesicle production in *E. coli* [14,19]. Thus, for $R_{c}\approx \text{16 nm}$, our model yields the observed increase in f(ρ) upon Lpp deletion. As illustrated in Fig 2(d), the quantitative predictions of Eq. (1) for the fold change in vesicle production with changes in the Lpp density depend on the specific value of $R_{c}$ used. To test our model predictions in Fig 2(d), and to directly estimate $R_{c}$ from experiments on vesicle formation, we developed an experimental system that allows quantitative measurement of vesicle production in *E. coli* as a function of the Lpp density, which we describe next.

### Testing predictions of bacterial vesicle production

**Fig 3:**
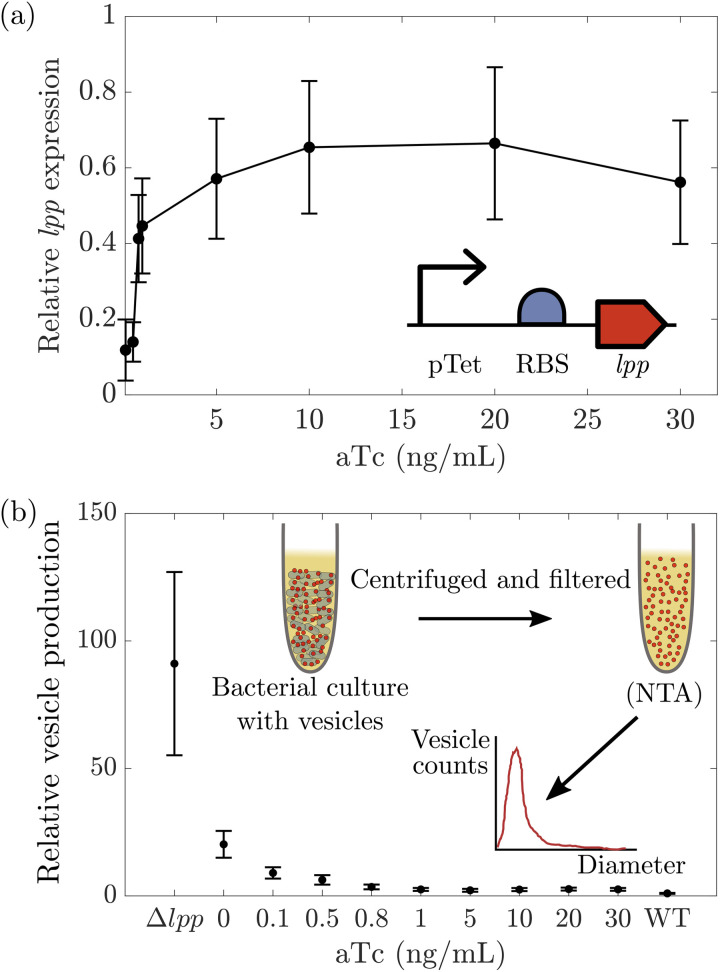
Experimental measurement of the change in bacterial vesicle production with *lpp* expression level. (a) A genetic circuit to control the expression of *lpp* was inserted into an *E. coli* strain with the native copy of *lpp* deleted. The expression level of *lpp* was dependent on the concentration of the inducer, aTc, added to the cell culture. (b) Vesicles were separated from cells via centrifugation and filtering. The density of vesicles produced at each inducer concentration was determined by NTA. Error bars represent standard error; n≥3.

To experimentally test our model predictions, we introduced a plasmid expressing *lpp* under an inducible promoter to an *E. coli* strain lacking the native copy of *lpp* [see Fig 3(a)]. In this strain, the number of Lpp proteins present in each cell was dependent on the concentration of the inducer anhydrotetracycline, aTc. Cultures were grown at 37 °C in Lysogeny broth to exponential phase with variable concentrations of inducer. Lpp expression levels relative to WT were estimated using a combination of qPCR targeting the *lpp* mRNA and a GFP reporter strain utilizing the same promoter [see Methods]. First, the expression level of *lpp* from the plasmid relative to expression from the native promoter in the genome was determined via qPCR for stationary phase cells induced at 5 ng/mL aTc. These measurements revealed that *lpp* was expressed at ~20% (0.202±0.066) of WT when induced at this level. The GFP reporter strain was then used to determine the relative response of the plasmid to induction across a range of aTc concentrations by measuring the normalized fluorescence [see S4 Fig]. Using qPCR, we estimated the conversion factor between GFP fluorescence and the relative expression of *lpp*, at 5ng/mL aTc. We found that at 5 ng/mL aTc, the GFP strain produced ~250 arbitrary fluorescence units (afu), yielding a conversion factor of $\text{8}\times {\text{10}}^{-\text{4}}\;\%$ of WT *lpp* expression per afu. Application of this same conversion factor to other induction levels can be complicated by varying levels of translation efficiency and protein degradation between GFP and Lpp. However, these complications are not expected to affect the estimated fold changes in gene expression or protein production [see S2 Text], making a single conversion factor sufficient. We therefore applied the same conversion factor to all GFP fluorescence measurements, and thus estimated the relative *lpp* expression levels at the measured induction levels in both exponential phase and stationary phase cultures. We took these estimates of relative *lpp* expression as a proxy for the relative Lpp density [see S2 Text]. With high levels of induction, we were able to restore the *lpp* expression level to approximately ~70% of that found in WT cells. Expression of *lpp* from the plasmid restored cellular shape to the WT appearance, indicating a phenotypic response to *lpp* expression in our strain [see S5 Fig].

Using the experimental system in Fig 3(a), the density of Lpp proteins could be tuned to examine vesicle production over a range of *lpp* expression levels [see Fig 3(b)]. To quantify vesicle production, vesicles were first harvested from exponential phase cultures. Cells were then pelleted via centrifugation, and the culture supernatant was filtered through a 0.22 micron filter. The concentration of vesicles produced by each culture was measured via nanoparticle tracking analysis (NTA). In NTA, deflection of laser light off of nanoscale objects under flow enables quantification of the number and size of such objects within a sample. NTA is commonly used to measure the concentration of vesicles [40,41]. Vesicle concentrations at each induction level were normalized to cell density, as determined by colony counting. As shown in Fig 3(b), reduced levels of *lpp* expression consistently increase vesicle production, up to a fold change of nearly 100 for the *Δlpp* strain. This increase in vesicle production of roughly two orders of magnitude in exponential phase cells is consistent with the increase seen in previous measurements of vesicle production taken during stationary phase in *Δlpp* strains [14,19]. Conversely, when *lpp* was expressed at ~70% of WT levels, vesicle production was reduced to nearly WT levels, indicating proper functioning of Lpp when expressed from the plasmid [see Fig 3(b)].

We also measured vesicle production in stationary phase cultures, a growth condition commonly used in previous experimental studies of bacterial vesicles [14,18,42]. As demonstrated in S6 Fig, vesicle production in stationary phase did not show a consistent increase with reduced levels of Lpp induction. In some cases, vesicle production for similar levels of *lpp* expression differed by an order of magnitude. Furthermore, vesicle production did not return to WT levels when *lpp* was expressed from the plasmid at nominally WT levels. These results suggest that stationary phase cultures yield complications with Lpp expression from the plasmid used here. We therefore focused on *E. coli* in exponential phase cultures.

**Fig 4:**
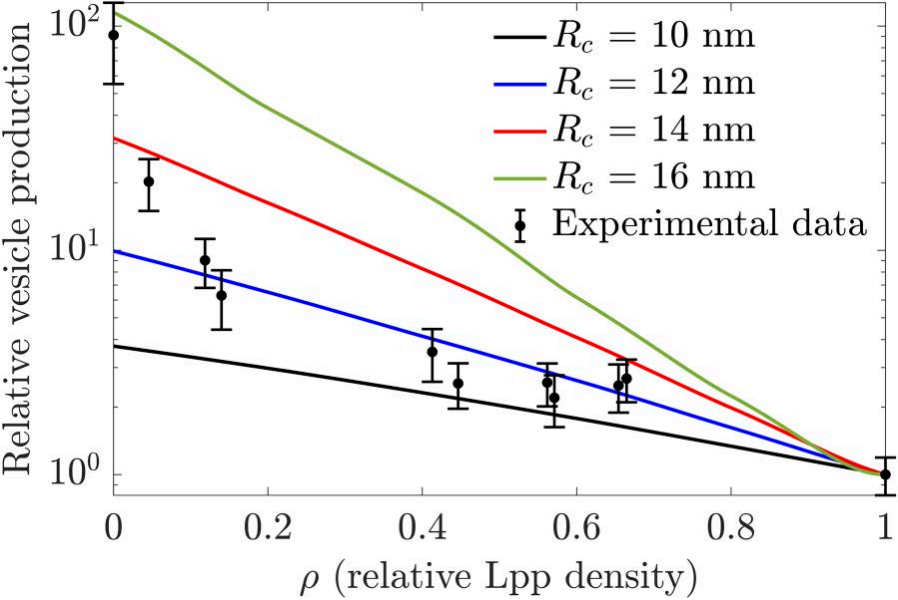
Comparison of model predictions and experimental measurements. Experimentally measured fold changes in bacterial vesicle number with respect to WT *E. coli* as a function of Lpp density (data points) and corresponding model predictions reproduced from Fig 2(d) (solid curves). Error bars represent standard error; n≥3.

Upon normalizing the vesicle concentration measured at a given Lpp expression level with respect to the vesicle concentration measured for WT cells, direct quantitative comparisons can be made between experimental results and model predictions. Fig 4 shows that, as predicted by our model in Eq. (1), bacterial vesicle production in *E. coli* increases approximately exponentially with decreasing ρ for most Lpp densities measured in our experiments. Most measured fold changes in vesicle production align closely with model results obtained with $R_{c}\approx \text{12}$ nm. As detailed in the previous two sections, the specific numerical values of $R_{c}$ extracted from Eq. (1) depend on the specific model formulation used, with different model formulations typically yielding $R_{c}$ for the data in Fig 4 that differ by a few nanometers [see supplemental S1, S2, and S3 Figs, and S1 Text]. In particular, the specific numerical values of $R_{c}$ implied by the model formulation used here should be viewed as lower bounds on $R_{c}$. Fig 4 shows that the simple physical model of bacterial vesicle formation in Eq. (1), which only involves a single parameter $R_{c}$, can capture quantitatively bacterial vesicle production for most measured crosslink densities.

Note from Fig 4 that, if $R_{c}$ is constant with the Lpp density, our model in Eq. (1) fails to capture the rapid increase in vesicle production observed at low Lpp densities, with no single choice of critical radius capturing the behavior at both low and intermediate Lpp densities. For instance, as already pointed out in the context of Fig 2(d), a critical radius $R_{c}=\text{16}$ nm yields an increase in vesicle production at ρ=0 consistent with our as well as previous experiments [14,19], but Fig 4 shows that $R_{c}=\text{16}$ nm fails to capture experimental results for intermediate Lpp densities. This discrepancy between experimental results and model predictions suggests a breakdown in our model assumptions as Lpp becomes severely depleted. In the supplemental information S3 Text, S7 Fig, and S8 Fig we show that if $R_{c}$ is allowed to decrease somewhat with the Lpp density, our model can capture the data in Fig 4 for all measured Lpp densities.

## Discussion

We have developed and tested a simple physical model of vesicle production in Gram-negative bacteria. Our model assumes that the density of proteins crosslinking the outer membrane and the cell wall is a crucial determinant of bacterial vesicle production. In particular, building on prior experiments on vesicle formation in bacteria [19,27], we assume that vesicles form in untethered outer membrane regions that lack connections between the outer membrane and the cell wall. Furthermore, our model assumes that vesicles form primarily in untethered membrane regions with radii greater than some critical value, $R_{c}$. As the density of crosslinking proteins decreases, and the sizes of untethered membrane regions increase, the model predicts an increase in vesicle production. We validated our model against experimental measurements of vesicle production in *E. coli* under varying expression levels of Lpp, the most prevalent outer membrane crosslinking protein in *E. coli*. We find that, for most measured Lpp densities, our model successfully predicts the observed fold changes in vesicle production. While the precise value of $R_{c}$ depends on the model formulation used, our experiments roughly indicate ${\text{10 nm}≲R}_{c}≲\text{20 nm}$.

The essential predictions of our model are robust with respect to changes in our estimates of the WT crosslink number, as well as to changes in the spatial arrangement of crosslinks. We focused here on a particularly simple model formulation, which suggested $R_{c}\approx \text{12 nm}$ for most measured Lpp densities. We estimated the total number of Lpp crosslinks in the outer membrane from an upper bound on the Lpp copy number in *E. coli*, and assumed that one-third of Lpp proteins would form crosslinks. We furthermore assumed that all OmpA form dimers, that all OmpA dimers form crosslinks, and that all Pal proteins form crosslinks. These assumptions almost certainly overrepresent the number of OmpA and Pal crosslinks as, for instance, Pal has been reported to interact favorably with Lpp, OmpA, and TolB [33,37]. Furthermore, it is possible that we have underestimated the fraction of Lpp that form crosslinks, though experiments have indicated that a significant fraction of Lpp – approximately two-third – exist in an unbound configuration [43]. Reducing the total number of crosslinks in our model effectively increases the value of $R_{c}$ required to reproduce our experimental results. Conversely, increasing the total number of crosslinks decreases the value of $R_{c}$ required to capture our experimental data. For instance, we find that a 20% increase in the number of Lpp crosslinks decreases the value of $R_{c}$ in our model from $R_{c}\approx \text{12 nm}$ to $R_{c}\approx \text{11 nm}$, while a 20% decrease in the number of Lpp crosslinks increases $R_{c}$ to $R_{c}\approx \text{13 nm}$, without changing the qualitative predictions of our model [see S1 Fig].

Furthermore, based on experiments suggesting that during normal cell growth crosslinks show no preference for any particular location on the outer membrane [38], we focused here on scenarios with no correlations among crosslink placements. Recent AFM experiments have suggested, however, that crosslinks can form clusters [39]. We find that attractive interactions between crosslinks tend to increase the critical radius implied by our model. Accounting for these effects would yield critical radii for vesicle formation closer to 20 nm. As more data on the number and spatial organization of crosslinks in the outer membrane become available, our model could be extended to more strongly constrain $R_{c}$.

For vesicle formation stemming from bulging of the outer membrane, there is an energetic cost associated with membrane deformations. What might drive such bulge formation? We can address this question by drawing an analogy to bulge formation in cytoplasmic membranes. Starting from basic principles of membrane mechanics, it was found previously that a cytoplasmic membrane pressed against the cell wall forms a bulge if the cell wall has a pore with a radius above some critical value [44],


$$\begin{array}{c} R_{c}\approx \text{2 }{\left(\frac{\kappa }{P}\right)}^{\text{1}/\text{3}}\;, \end{array}$$ (2)

where κ denotes the bending rigidity of the membrane and P denotes the turgor pressure across the membrane. Membrane patches covering pores with radii ${R>R}_{c}$ are expected to be unstable to bleb formation [44]. Equation (2) predicts that bulge formation is opposed by the energy cost of membrane bending, which increases $R_{c}$, and facilitated by turgor pressure, which decreases $R_{c}$. In subsequent work it was found that entropic and stretching energies may also play important roles in the energetics of membrane bulging [45]. Equation (2) was found to capture basic features of membrane bulging in Gram-negative bacteria [44]. Similar considerations also apply to Gram-positive bacteria, which show a thicker cell wall [46]. In particular, an expression similar to Eq. (2) can be derived for Gram-positive bacteria, which correctly predicts the minimum pore size in the cell walls of lysed, Gram-positive cells [46].

How does the simple estimate in Eq. (2) compare with our experimental estimates of $R_{c}$? As noted above, our results suggest the approximate range ${\text{10 nm}≲R}_{c}≲\text{20 nm}$. Prior estimates of $R_{c}$ for cytoplasmic membranes yielded $R_{c}\approx \text{18 nm}$ [44] and ${\text{15 nm}≲R}_{c}≲\text{24 nm}$ [46] for membrane bulge formation in Gram-negative and Gram-positive bacteria, respectively. Even though the systems studied here and in refs. [44,46] are somewhat different, these estimates are consistent with our results. There is considerable uncertainty regarding the values of κ and P in Eq. (2) relevant for bacterial outer membranes. However, by assuming particular ranges of κ and P, we can use Eq. (2) to make broad estimates of $R_{c}$. For *E. coli*, available estimates of the outer membrane bending rigidity suggest $\text{10 }k_{B}T≲\kappa ≲\text{100 }k_{B}T$ [47]. The total turgor pressure across the *E. coli* cell envelope is expected to lie typically between 0.3 atm and 3 atm [48,49]. It is unclear what percentage of this pressure differential occurs across the outer membrane. Applying the range 0.3 atm≲P≲3 atm to the outer membrane, we find from Eq. (2) the broad range ${\text{10 nm}≲R}_{c}≲\text{50 nm}$, which is consistent with our experiments on vesicle formation. Thus, Eq. (2) seems to capture the approximate range of $R_{c}$ extracted from our experiments.

The above estimates suggest that, while bacterial outer membranes have a highly complex structure, a basic aspect of bacterial vesicle formation may be captured by a competition between bending forces and turgor pressure. We note, however, that Eq. (2) was originally developed to describe large membrane bulges with diameters on the order of 1 µm, while the bacterial vesicles examined in this study have diameters on the order of 100 nm (see S9 Fig), a feature that was consistent across all experimental conditions, with WT cells having a mode diameter of 88 nm and Δlpp cells having a mode diameter of 85 nm. Thus, the observed vesicle radii are comparable to, but somewhat larger than, the values of $R_{c}$ implied by our model. In future work, it would be interesting to quantify the relation between the size of untethered outer membrane regions, the membrane mechanical properties of the outer membrane, and the size of bacterial vesicles.

Assuming a constant critical radius for vesicle formation, our model successfully predicts *E. coli* vesicle production in exponential phase at most induced expression levels of Lpp, but fails to predict the sharp increase in vesicle production observed at low Lpp densities. We suggest that this discrepancy between theory and experiment can be explained based on a decrease in $R_{c}$ at low Lpp levels. In light of Eq. (2) such a decrease in the critical radius may be interpreted as a decrease in the outer membrane bending rigidity or an increase in the turgor pressure across the outer membrane. It is unclear how a reduction in the Lpp density would result in a sharp increase in turgor pressure across a narrow range of Lpp densities, which would be required to capture the measured vesicle production at low Lpp densities. Interestingly, however, it has been found that Lpp contributes substantially to the outer membrane stiffness [23] and the overall cell stiffness [50,51]. We find that, via Eq. (2), a decrease in the outer membrane bending rigidity at low Lpp densities can indeed account for the observed increase in vesicle production. These results are consistent with previous experiments on vesicle production in an *E. coli* strain in which the Lpp domain responsible for the binding of Lpp to the cell wall was deleted, while presumably retaining WT levels of Lpp in the outer membrane [19]. In this strain, the outer membrane bending rigidity and, hence, the critical radius for vesicle formation would be expected to be approximately the same as in WT *E. coli*. Our model predicts a fold change in vesicle production ~10 at ρ=0 for the value $R_{c}\approx \text{12 nm}$ suggested by our measurements of vesicle production at intermediate Lpp densities, which indeed matches the fold change in vesicle production observed in Ref. [19].

As more quantitative data on the physical properties of the bacterial outer membrane become available, and estimates of $R_{c}$ using this data become more precise, our model could be used as a framework for predicting the crosslink binding frequency and the crosslink organization, which may have interesting implications for the dynamics of vesicle formation. In particular, Lpp exists in a dynamic equilibrium between its free and bound forms, regularly binding and unbinding from the cell wall [52]. Interestingly, physical theory and simulations have suggested that crosslinks connecting a thermally fluctuating membrane to a rigid substrate will tend to form clusters, with membrane regions between these clusters being able to deform more freely [53]. As more data on the crosslink number and organization become available, this data could be employed to further constrain and test our model. Our results suggest that systematic changes in the Lpp density could be used to probe the dynamic interplay between crosslink binding and unbinding, crosslink organization, and outer membrane deformation.

Our physical model of bacterial vesicle formation produces quantitative agreement with experiments in *E. coli* while only involving a few basic assumptions, making it potentially applicable to a wide range of bacterial species. Our model thus suggests avenues for the systematic modification of bacterial vesicle production and outer membrane stability in *E. coli* as well as other Gram-negative bacteria. For instance, the framework developed here could be used to predict and quantify the modulation of bacterial vesicle production due to changes in the osmotic environment of bacteria, the outer membrane bending rigidity and fluidity, and the density and organization of outer membrane crosslinking proteins.

## Methods

### Bacterial strains and growth conditions

For WT *E. coli*, we used here the strain *Escherichia coli* (K-12 MG1655). For the *lpp* deletion strain, we used the strain *Escherichia coli* (K12-BW25113) acquired from the Keio knockout collection [54]. *E. coli* DH5a was used for plasmid construction. The strain with variable expression of *lpp* was constructed via electroporation of a plasmid with tetR-controlled expression of the *lpp* gene into the *lpp* deletion strain. The strain with variable expression of GFP was constructed via electroporation of a plasmid with tetR-controlled expression of GFP into MG1655.

Strains were inoculated from frozen stocks and cultured in 5 mL of lysogeny broth (LB from BD Difco) contained in 14 mL Falcon tubes. 100 µg/mL carbenicillin or 50 µg/mL kanamycin were added as appropriate for plasmid maintenance and to prevent contamination. Initial cultures were inoculated from frozen stocks and grown overnight while shaking at 200 rpm and 37 °C. Overnight cultures were then diluted 1:100 in fresh media, anhydrotetracycline (aTc from Sigma-Aldrich) was added for experiments that required induction of *lpp* expression, and cells were grown to late exponential phase, OD of ~0.8. aTc stocks at 200 ng/µL were prepared by dissolving aTc in ethanol.

### Plasmid construction

The plasmid with tetR-controlled expression of the *lpp* gene was constructed via Gibson assembly [see S1 Table]. pDSG372, which contains tetR, was combined with a plasmid backbone with an SC101 origin. The *lpp* gene was acquired from the *E. coli* genome. The *lpp* or *gfp* gene was added after the pTet promoter in the resulting pTet-lpp plasmid via Gibson assembly to control *lpp* or *gfp* expression with an aTc inducer. New constructs were verified via Sanger sequencing.

### Vesicle harvesting and quantification

Following cell growth, cultures were spun down for 30 minutes at 4200 rpm and 4 °C. The supernatant, which contains the extracellular vesicles, was extracted and run through a 0.22 micron PTFE filter (VWR). Filtered supernatant was collected and saved for vesicle characterization. All vesicle samples were stored at 4 °C and examined within 48 hours of isolation.

Vesicle sizes and counts were quantified using NTA on a Malvern Instruments NanoSight NS300. Data were collected as three one-minute videos, with a detection threshold of 10. Measurements were performed on filtered supernatant or filtered supernatant diluted in deionized water. Particles were diluted immediately before measurement such that there were between 10 and 100 particles/frame. In prior work, filtered supernatant was processed further through multiple steps of washing and ultracentrifugation in order to concentrate and purify the vesicles for transfer experiments [55]. As shown in S9 Fig, additional centrifugation steps only resulted in minor losses of vesicles and did not impact the size distribution of the detected vesicles. In our protocol we therefore omitted centrifugation steps prior to analysis. Vesicle counts were normalized with respect to the cell density, which was measured via colony counting. Fold changes in vesicle production were calculated through comparison to vesicle production in WT cells with *lpp* expressed at WT levels from the genome.

### Quantification of relative *lpp* expression

To quantify the expression of *lpp* in our strain, we used a combination of quantitative PCR (qPCR) and fluorescent reporter constructs. First, the expression level of *lpp* from the plasmid relative to expression from the native promoter in the genome was determined via qPCR. We used primers targeting the *lpp* gene, with *idnT* as a reference gene [56] [see S2 Table for primer sequences]. Total RNA was extracted from stationary phase cultures of *E. coli* using a T2010 miniprep kit (New England Biolabs) and processed using the Luna Universal One-Step RT-qPCR kit (New England Biolabs). qPCR measurements were performed using an Agilent AriaMx Real-time PCR system. qPCR data were processed following the procedure detailed in Ref. [57] to determine the fluorescence baselines and efficiencies for each amplicon as well as the ideal threshold fluorescence for determining crossing points. All efficiencies used in our calculations were within the recommended range of 1.8–2.1. Relative *lpp* expression levels were then calculated following the procedure in Ref. [57]. *lpp* expression was measured with n≥4 for *E. coli* MG1655 and *E. coli* with plasmid pTet-lpp induced at 5 ng/mL aTc.

To extend the calibration curve to a wider range of aTc induction concentrations, gene expression was measured via a GFP reporter construct. A version of the pTet-lpp plasmid was constructed substituting *gfp* for the *lpp* gene. *gfp* expression was induced at aTc concentrations from 0 to 100 ng/mL, and fluorescence was measured in a Tecan infinite m200pro 96 well plate reader. Background fluorescence was subtracted, and fluorescence measurements were normalized to OD_600._ Relative fluorescence was taken as a proxy for relative expression of *gfp.* We then determined the conversion factor between relative GFP fluorescence and relative *lpp* expression by calculating the ratio of the relative fluorescence in our GFP reporter strain and the qPCR measurement of *lpp* expression in our *lpp* strain, both induced at 5 ng/mL aTc in stationary phase. By applying this conversion factor to the remaining fluorescent reporter measurements in stationary phase and exponential phase, expression of *lpp* relative to the WT strain was determined as reported in Fig 3(a), S4b Fig, S4d Fig, and S6a Fig.

### Estimating the crosslink number

In our model of bacterial vesicle formation, we consider four types of crosslinks: Lpp, Pal, OmpA, and Lpp-OmpA. Pal crosslinks are a single Pal protein. Lpp crosslinks are trimers composed of three Lpp proteins. OmpA crosslinks are dimers composed of two OmpA proteins. We model Lpp-OmpA crosslinks as an Lpp trimer bound to an OmpA dimer.

References [22,32,33] suggest that there can be up to 750,000 Lpp, 100,000 OmpA, and 20,000 Pal proteins per WT *E. coli* cell. We assume that every Pal forms a crosslink and that all OmpA dimerize [36]. Furthermore, we assume that all Lpp form trimers, and that only 1/3 of Lpp trimers form crosslinks [34,35]. Reference [36] suggests that approximately 3/4 of all OmpA dimer crosslinks form a complex with an Lpp trimer crosslink. At WT Lpp densities, this corresponds to roughly 1/2 of all Lpp trimer crosslinks forming a complex with an OmpA dimer crosslink. We thus estimate that there are up to 42,000 Lpp crosslinks, 42,000 Lpp-OmpA crosslinks, 8000 OmpA crosslinks, and 20,000 Pal crosslinks per WT *E. coli* cell. We adjust these estimates as the Lpp density is reduced relative to WT *E. coli*, assuming that 1/2 of all Lpp trimer crosslinks form a complex with an OmpA dimer crosslink at all Lpp densities. When the Lpp density is reduced to 0, we have 50,000 OmpA crosslinks and 20,000 Pal crosslinks per *E. coli* cell.

We assume an *E. coli* outer membrane area equal to $\text{6 }\mu {\text{m}}^{\text{2}}$, resulting in a total of ~185 crosslinks per 100 nm× 100 nm outer membrane patch at WT Lpp densities, ρ=1, and ~115 crosslinks per 100 nm× 100 nm outer membrane patch when the Lpp density is reduced to zero. Assuming that the average spacing between crosslinks is approximately equal to the square root of the inverse crosslink density, we have the average spacings 7 nm and 9 nm at ρ=1 and ρ=0, respectively. While we fixed here the area of the *E. coli* outer membrane to the constant value $\text{6 }\mu {\text{m}}^{\text{2}}$, a more detailed model could more precisely quantify the *E. coli* outer membrane area as a function of the Lpp density.

### Characterization of untethered outer membrane regions

For each outer membrane patch considered in our model, we generated a Voronoi diagram using the spatial coordinates of the crosslinks in the membrane patch. The vertices of these Voronoi diagrams represent the circumcenters of each neighboring triplet of crosslinks. In this way, triplets of points are used to uniquely define circular regions in the membrane patch under consideration. To avoid boundary effects, our calculations excluded circular membrane regions for which any portion of the circle extended beyond the boundaries of the membrane patch under consideration. We tested larger membrane patch sizes to ensure that the finite size of the membrane patches used here did not yield artifacts in the R-distributions of untethered membrane regions. To generate R-distributions, we averaged over 100,000 membrane patches at each crosslink density considered here. The R-distributions described here represent kernel densities of the radii of untethered membrane patches scaled to the outer membrane area of $\sim \text{6 }\mu {\text{m}}^{\text{2}}$ associated with *E. coli*.

### Fold change predictions

To generate a curve predicting the fold changes in bacterial vesicle production for a particular choice of the critical radius $R_{c}$, we considered membrane patches with Lpp densities between ρ=0 and ρ=1 at intervals of Δρ=0.1. For each of these ρ values, we computed the number of untethered membrane regions with ${R>R}_{c}$. To estimate the number of untethered membrane regions with ${R>R}_{c}$ at values of ρ within these discrete ρ-intervals, we performed a cubic splines interpolation using the *interp1* function in MATLAB (version R2022a). We thus considered ρ between ρ=0 and ρ=1 at intervals of δρ=0.001. Fold changes in vesicle production were calculated by normalizing the number of untethered membrane regions with ${R>R}_{c}$ at a given ρ with respect to the corresponding value at ρ=1, as in Eq. (1).

## Supporting information

S1 Text Model predictions with alternative propensity functions. (PDF)

S2 Text Fold change in steady state Lpp or GFP with protein degradation or mRNA degradation. (PDF)

S3 Text Variation of the critical radius with Lpp density. (PDF)

S1 Fig Model predictions with modified crosslink numbers. (PDF)

S2 Fig Model predictions under attractive or repulsive crosslink interactions. (PDF)

S3 Fig Model predictions with alternative propensity functions. (PDF)

S4 Fig Fluorescence curves and relative *lpp* expression. (PDF)

S5 Fig *E. coli* aspect ratio is restored to WT values at high enough levels of plasmid induction. (PDF)

S6 Fig Stationary phase measurements of vesicle production. (PDF)

S7 Fig Variation of the critical radius with Lpp density due to varying membrane bending rigidity. (PDF)

S8 Fig Variation of the critical radius with Lpp density due to varying turgor pressure. (PDF)

S9 Fig Ultracentrifugation has a minimal effect on vesicle size and concentration. (PDF)

S1 Table qPCR primers used in this study. (PDF)

S2 Table Plasmid primers used in this study. (PDF)

## References

[1] Brown L, Wolf JM, Prados-Rosales R, Casadevall A. Through the wall: extracellular vesicles in Gram-positive bacteria, mycobacteria and fungi. Nat Rev Microbiol. 2015;13(10):620–30. doi: 10.1038/nrmicro3480 26324094 PMC4860279

[2] Kuehn MJ, Kesty NC. Bacterial outer membrane vesicles and the host-pathogen interaction. Genes Dev. 2005;19(22):2645–55. doi: 10.1101/gad.1299905 16291643

[3] Biller SJ, Schubotz F, Roggensack SE, Thompson AW, Summons RE, Chisholm SW. Bacterial Vesicles in Marine Ecosystems. Science. 2014;343: 183–186. doi: 10.1126/science.1245871 24408433

[4] Caruana JC, Walper SA. Bacterial Membrane Vesicles as Mediators of Microbe - Microbe and Microbe - Host Community Interactions. Front Microbiol. 2020;11:432. doi: 10.3389/fmicb.2020.00432 32265873 PMC7105600

[5] Bitto NJ, Chapman R, Pidot S, Costin A, Lo C, Choi J, et al. Bacterial membrane vesicles transport their DNA cargo into host cells. Sci Rep. 2017;7(1):7072. doi: 10.1038/s41598-017-07288-4 28765539 PMC5539193

[6] Toyofuku M. Bacterial communication through membrane vesicles. Biosci Biotechnol Biochem. 2019;83(9):1599–605. doi: 10.1080/09168451.2019.1608809 31021698

[7] Brameyer S, Plener L, Müller A, Klingl A, Wanner G, Jung K. Outer Membrane Vesicles Facilitate Trafficking of the Hydrophobic Signaling Molecule CAI-1 between Vibrio harveyi Cells. J Bacteriol. 2018;200(15):e00740–17. doi: 10.1128/JB.00740-17 29555694 PMC6040191

[8] Mashburn LM, Whiteley M. Membrane vesicles traffic signals and facilitate group activities in a prokaryote. Nature. 2005;437(7057):422–5. doi: 10.1038/nature03925 16163359

[9] Tran F, Boedicker JQ. Genetic cargo and bacterial species set the rate of vesicle-mediated horizontal gene transfer. Sci Rep. 2017;7(1):8813. doi: 10.1038/s41598-017-07447-7 28821711 PMC5562762

[10] Manning AJ, Kuehn MJ. Contribution of bacterial outer membrane vesicles to innate bacterial defense. BMC Microbiol. 2011;11(1):258. doi: 10.1186/1471-2180-11-258 22133164 PMC3248377

[11] McBroom AJ, Kuehn MJ. Release of outer membrane vesicles by Gram-negative bacteria is a novel envelope stress response. Mol Microbiol. 2007;63(2):545–58. doi: 10.1111/j.1365-2958.2006.05522.x 17163978 PMC1868505

[12] Turnbull L, Toyofuku M, Hynen AL, Kurosawa M, Pessi G, Petty NK, et al. Explosive cell lysis as a mechanism for the biogenesis of bacterial membrane vesicles and biofilms. Nat Commun. 2016;7:11220. doi: 10.1038/ncomms11220 27075392 PMC4834629

[13] Toyofuku M, Nomura N, Eberl L. Types and origins of bacterial membrane vesicles. Nat Rev Microbiol. 2019;17(1):13–24. doi: 10.1038/s41579-018-0112-2 30397270

[14] Kulp AJ, Sun B, Ai T, Manning AJ, Orench-Rivera N, Schmid AK, et al. Genome-wide assessment of outer membrane vesicle production in Escherichia coli. PLoS One. 2015;10(9):e0139200. doi: 10.1371/journal.pone.0139200 26406465 PMC4583269

[15] Nasu H, Shirakawa R, Furuta K, Kaito C. Knockout of mlaA increases Escherichia coli virulence in a silkworm infection model. PLoS One. 2022;17(7):e0270166. doi: 10.1371/journal.pone.0270166 35830444 PMC9278758

[16] Mashburn-Warren L, Howe J, Garidel P, Richter W, Steiniger F, Roessle M, et al. Interaction of quorum signals with outer membrane lipids: insights into prokaryotic membrane vesicle formation. Mol Microbiol. 2008;69(2):491–502. doi: 10.1111/j.1365-2958.2008.06302.x 18630345 PMC2615190

[17] Florez C, Raab JE, Cooke AC, Schertzer JW. Membrane distribution of the pseudomonas quinolone signal modulates outer membrane vesicle production in Pseudomonas aeruginosa. mBio. 2017;8:10. doi: 10.1128/mbio.01034-17 PMC5550756 28790210

[18] Tran F, Gangan MS, Weaver BP, Boedicker JQ. Membrane-binding biomolecules influence the rate of vesicle exchange between bacteria. Appl Environ Microbiol. 2022;88(23):e0134622. doi: 10.1128/aem.01346-22 36342184 PMC9746307

[19] Schwechheimer C, Sullivan CJ, Kuehn MJ. Envelope control of outer membrane vesicle production in Gram-negative bacteria. Biochemistry. 2013;52(18):3031–40. doi: 10.1021/bi400164t 23521754 PMC3731998

[20] Braun V, Wolff H. The murein-lipoprotein linkage in the cell wall of Escherichia coli. Eur J Biochem. 1970;14(2):387–91. doi: 10.1111/j.1432-1033.1970.tb00301.x 4918558

[21] Magnet S, Bellais S, Dubost L, Fourgeaud M, Mainardi J-L, Petit-Frère S, et al. Identification of the L,D-transpeptidases responsible for attachment of the Braun lipoprotein to Escherichia coli peptidoglycan. J Bacteriol. 2007;189(10):3927–31. doi: 10.1128/JB.00084-07 17369299 PMC1913343

[22] Li G-W, Burkhardt D, Gross C, Weissman JS. Quantifying absolute protein synthesis rates reveals principles underlying allocation of cellular resources. Cell. 2014;157(3):624–35. doi: 10.1016/j.cell.2014.02.033 24766808 PMC4006352

[23] Rojas ER, Billings G, Odermatt PD, Auer GK, Zhu L, Miguel A, et al. The outer membrane is an essential load-bearing element in Gram-negative bacteria. Nature. 2018;559(7715):617–21. doi: 10.1038/s41586-018-0344-3 30022160 PMC6089221

[24] Guo MS, Updegrove TB, Gogol EB, Shabalina SA, Gross CA, Storz G. MicL, a new σE-dependent sRNA, combats envelope stress by repressing synthesis of Lpp, the major outer membrane lipoprotein. Genes Dev. 2014;28(14):1620–34. doi: 10.1101/gad.243485.114 25030700 PMC4102768

[25] Winkle M, Hernández-Rocamora VM, Pullela K, Goodall ECA, Martorana AM, Gray J, et al. DpaA Detaches Braun’s Lipoprotein from Peptidoglycan. mBio. 2021;12(3):e00836-21. doi: 10.1128/mBio.00836-21 33947763 PMC8263019

[26] Bahadur R, Chodisetti PK, Reddy M. Cleavage of Braun’s lipoprotein Lpp from the bacterial peptidoglycan by a paralog of l,d-transpeptidases, LdtF. Proc Natl Acad Sci U S A. 2021;118(19):e2101989118. doi: 10.1073/pnas.2101989118 33941679 PMC8126863

[27] Deatherage BL, Lara JC, Bergsbaken T, Rassoulian Barrett SL, Lara S, Cookson BT. Biogenesis of bacterial membrane vesicles. Mol Microbiol. 2009;72(6):1395–407. doi: 10.1111/j.1365-2958.2009.06731.x 19432795 PMC2745257

[28] Schwechheimer C, Kuehn MJ. Outer-membrane vesicles from Gram-negative bacteria: biogenesis and functions. Nat Rev Microbiol. 2015;13(10):605–19. doi: 10.1038/nrmicro3525 26373371 PMC5308417

[29] Arora A, Abildgaard F, Bushweller JH, Tamm LK. Structure of outer membrane protein A transmembrane domain by NMR spectroscopy. Nat Struct Biol. 2001;8(4):334–8. doi: 10.1038/86214 11276254

[30] Abergel C, Walburger A, Chenivesse S, Lazdunski C. Crystallization and preliminary crystallographic study of the peptidoglycan-associated lipoprotein from Escherichia coli. Acta Crystallogr D Biol Crystallogr. 2001;57(Pt 2):317–9. doi: 10.1107/s0907444900019739 11173492

[31] Shu W, Liu J, Ji H, Lu M. Core structure of the outer membrane lipoprotein from *Escherichia coli* at 1.9 A resolution. J Mol Biol. 2000;299(4):1101–12. doi: 10.1006/jmbi.2000.3776 10843861

[32] Koebnik R, Locher KP, Van Gelder P. Structure and function of bacterial outer membrane proteins: barrels in a nutshell. Mol Microbiol. 2000;37(2):239–53. doi: 10.1046/j.1365-2958.2000.01983.x 10931321

[33] Cascales E, Bernadac A, Gavioli M, Lazzaroni J-C, Lloubes R. Pal Lipoprotein ofEscherichia coliPlays a Major Role in Outer Membrane Integrity. J Bacteriol. 2002;184(3):754–9. doi: 10.1128/jb.184.3.754-759.2002 11790745 PMC139529

[34] Choi DS, Yamada H, Mizuno T, Mizushima S. Trimeric structure and localization of the major lipoprotein in the cell surface of Escherichia coli. J Biol Chem. 1986;261(19):8953–7. doi: 10.1016/s0021-9258(19)84474-5 3013869

[35] Cowles CE, Li Y, Semmelhack MF, Cristea IM, Silhavy TJ. The free and bound forms of Lpp occupy distinct subcellular locations in Escherichia coli. Mol Microbiol. 2011;79(5):1168–81. doi: 10.1111/j.1365-2958.2011.07539.x 21219470 PMC3090202

[36] Samsudin F, Boags A, Piggot TJ, Khalid S. Braun’s Lipoprotein Facilitates OmpA Interaction with the Escherichia coli Cell Wall. Biophys J. 2017;113(7):1496–504. doi: 10.1016/j.bpj.2017.08.011 28978443 PMC5627309

[37] Bouveret E, Bénédetti H, Rigal A, Loret E, Lazdunski C. In vitro characterization of peptidoglycan-associated lipoprotein (PAL)-peptidoglycan and PAL-TolB interactions. J Bacteriol. 1999;181(20):6306–11. doi: 10.1128/JB.181.20.6306-6311.1999 10515919 PMC103764

[38] Hiemstra H, Nanninga N, Woldringh CL, Inouye M, Witholt B. Distribution of newly synthesized lipoprotein over the outer membrane and the peptidoglycan sacculus of an Escherichia coli lac-lpp strain. J Bacteriol. 1987;169(12):5434–44. doi: 10.1128/jb.169.12.5434-5444.1987 3316185 PMC213969

[39] Sheng Q, Zhang M-Y, Liu S-M, Chen Z-W, Yang P-L, Zhang H-S, et al. In situ visualization of Braun’s lipoprotein on E. coli sacculi. Sci Adv. 2023;9(3). doi: 10.1126/sciadv.add8659 PMC9858504 36662863

[40] Dragovic RA, Gardiner C, Brooks AS, Tannetta DS, Ferguson DJP, Hole P, et al. Sizing and phenotyping of cellular vesicles using Nanoparticle Tracking Analysis. Nanomedicine. 2011;7(6):780–8. doi: 10.1016/j.nano.2011.04.003 21601655 PMC3280380

[41] Gerritzen MJH, Martens DE, Wijffels RH, Stork M. High throughput nanoparticle tracking analysis for monitoring outer membrane vesicle production. J Extracell Vesicle. 2017;6(1). doi: 10.1080/20013078.2017.1333883 PMC5505008 28717425

[42] Schwechheimer C, Rodriguez DL, Kuehn MJ. NlpI-mediated modulation of outer membrane vesicle production through peptidoglycan dynamics in Escherichia coli. Microbiol Open. 2015;4(3):375–89. doi: 10.1002/mbo3.244 25755088 PMC4475382

[43] Inouye M, Shaw J, Shen C. The Assembly of a Structural Lipoprotein in the Envelope of *Escherichia coli*. J Biol Chem. 1972;247(24):8154–9. doi: 10.1016/s0021-9258(20)81822-5 4565677

[44] Daly KE, Huang KC, Wingreen NS, Mukhopadhyay R. Mechanics of membrane bulging during cell-wall disruption in gram-negative bacteria. Phys Rev E Stat Nonlin Soft Matter Phys. 2011;83(4 Pt 1):041922. doi: 10.1103/PhysRevE.83.041922 21599215 PMC12380458

[45] Wong F, Amir A. Mechanics and dynamics of bacterial cell lysis. Biophys J. 2019;116(12):2378–89. doi: 10.1016/j.bpj.2019.04.040 31174849 PMC6588734

[46] Mitchell GJ, Wiesenfeld K, Nelson DC, Weitz JS. Critical cell wall hole size for lysis in Gram-positive bacteria. J R Soc Interface. 2013;10(80):20120892. doi: 10.1098/rsif.2012.0892 23303219 PMC3565739

[47] Hsu P-C, Samsudin F, Shearer J, Khalid S. It is complicated: curvature, diffusion, and lipid sorting within the two membranes of Escherichia coli. J Phys Chem Lett. 2017;8(22):5513–8. doi: 10.1021/acs.jpclett.7b02432 29053278

[48] Cayley DS, Guttman HJ, Record MT Jr. Biophysical characterization of changes in amounts and activity of Escherichia coli cell and compartment water and turgor pressure in response to osmotic stress. Biophys J. 2000;78(4):1748–64. doi: 10.1016/s0006-3495(00)76726-9 10733957 PMC1300771

[49] Deng Y, Sun M, Shaevitz JW. Direct measurement of cell wall stress stiffening and turgor pressure in live bacterial cells. Phys Rev Lett. 2011;107(15):158101. doi: 10.1103/PhysRevLett.107.158101 22107320

[50] Mathelié-Guinlet M, Asmar AT, Collet J-F, Dufrêne YF. Lipoprotein Lpp regulates the mechanical properties of the E. coli cell envelope. Nat Commun. 2020;11(1). doi: 10.1038/s41467-020-15489-1 PMC7156740 32286264

[51] Vadillo-Rodriguez V, Schooling SR, Dutcher JR. In Situ characterization of differences in the viscoelastic response of individual Gram-Negative and Gram-Positive Bacterial Cells. J Bacteriol. 2009;191(17):5518–25. doi: 10.1128/jb.00528-09 19581369 PMC2725611

[52] Liang Y, Hugonnet J-E, Rusconi F, Arthur M. Peptidoglycan-tethered and free forms of the Braun lipoprotein are in dynamic equilibrium in Escherichia coli. eLife. 2024;12. doi: 10.7554/elife.91598 PMC11449479 39360705

[53] Weikl TR, Lipowsky R. Adhesion-induced phase behavior of multicomponent membranes. Phys Rev E Stat Nonlin Soft Matter Phys. 2001;64(1 Pt 1):011903. doi: 10.1103/PhysRevE.64.011903 11461284

[54] Baba T, Ara T, Hasegawa M, Takai Y, Okumura Y, Baba M, et al. Construction of Escherichia coli K-12 in-frame, single-gene knockout mutants: the Keio collection. Mol Syst Biol. 2006;2:2006.0008. doi: 10.1038/msb4100050 16738554 PMC1681482

[55] Mosby CA, Perez Devia N, Jones MK. Comparison of Methods for Quantifying Extracellular Vesicles of Gram-Negative Bacteria. Int J Mol Sci. 2023;24(20):15096. doi: 10.3390/ijms242015096 37894776 PMC10606555

[56] Zhou K, Zhou L, Lim Q’En, Zou R, Stephanopoulos G, Too H-P. Novel reference genes for quantifying transcriptional responses of Escherichia coli to protein overexpression by quantitative PCR. BMC Mol Biol. 2011;12:18. doi: 10.1186/1471-2199-12-18 21513543 PMC3110127

[57] Ruijter JM, Ramakers C, Hoogaars WMH, Karlen Y, Bakker O, van den Hoff MJB, et al. Amplification efficiency: linking baseline and bias in the analysis of quantitative PCR data. Nucleic Acids Res. 2009;37(6):e45. doi: 10.1093/nar/gkp045 19237396 PMC2665230

