# Supplementary material for: The density of Braun’s Lipoprotein determines vesicle production in E. coli
Source: PLoS One. 2025 Sep 19;20(9):e0332156. doi: 10.1371/journal.pone.0332156 (PMC12448975; doi:10.1371/journal.pone.0332156)
Supplement: S1 Text — (PDF) [file pone.0332156.s001.pdf]

S1 Text. Model predictions with alternative propensity functions

In the main text we assumed that vesicle formation only occurs in untethered outer membrane regions greater than a critical size, and that all untethered outer membrane regions greater than that size have the same propensity for forming a vesicle. In Eq. (1) of the main text we represented this assumption mathematically through $w(R) = \theta(R - R_c)$, where $\theta(x)$ is the Heaviside step function. In this supplemental text, we explore how the predictions of our model change if we assume different functional forms of $w(R)$.

We first relax the assumption that all untethered membrane regions with radii R greater than the critical radius R_c have an equal propensity for forming a vesicle. Assume, for instance, that $w(R)$ shows a linear increase beyond the critical radius R_c,

$$w(R) = \alpha\,(R - R_c)\,\theta(R - R_c)\,. \tag{S1}$$

This form of $w(R)$ results in a weight of 0 for membrane patch radii R below R_c, and a weight that increases linearly with slope α for R greater than R_c, which is illustrated in S3 Fig(a) for $\alpha = 1$. Note that the parameter α cancels out in the fold change in Eq. (1) of the main text. We find that replacing $w(R) = \theta(R - R_c)$ by $w(R)$ in Eq. (S1) only has a minor impact on our model predictions [see S3 Fig(b)]. In particular, our qualitative model predictions are unchanged, while the value of R_c best matching our experimental measurements of bacterial vesicle production decreases slightly to $\sim$11 nm.

We next relax our assumption that vesicles can only form in untethered membrane regions greater than a critical size, by allowing for vesicle formation to occur with some non-zero propensity in untethered outer membrane regions with radii R smaller than R_c. In particular, consider a sigmoidal form of $w(R)$,

$$w(R) = \frac{\alpha}{1 + e^{-k\,(R - R_c)}}\,. \tag{S2}$$

Here, R_c defines the midpoint of the sigmoid, α defines the maximum weight, and k defines the steepness of the transition from $w = 0$ to $w = \alpha$. In S3 Fig(c) we show this form of $w(R)$ for several choices of R_c, with $\alpha = 1$ and $k = 1.5$. Note, again, that the parameter α cancels out in Eq. (1) of the main text. We see in S3 Fig(d) that with this form of $w(R)$ our predictions are qualitatively unchanged from the main text, while the value of R_c best matching our experimental measurements of bacterial vesicle production increases slightly to $\sim$14 nm.

The propensity functions in Eqs. (S1) and (S2) assume that the relative contributions of large, untethered membrane regions to bacterial vesicle formation are approximately constant or show a gradual increase with increasing R. To relax this assumption, consider

$$w(R) = \alpha\, e^{k\,R}\,. \tag{S3}$$

Here, α defines the propensity for $R = 0$ and k defines how quickly the propensity increases with increasing R. In S3 Fig(e) we plot $w(R)$ in Eq. (S3) with $\alpha = 1$ for several choices of k. Again, α

cancels out in Eq. (1) of the main text. For the R-range relevant for the experiments in the main text, the relative contributions of large, untethered membrane regions to bacterial vesicle formation can now differ by orders of magnitude. Intriguingly, this choice of $w(R)$ again produces fold change predictions for bacterial vesicle formation that are similar to those produced by the Heaviside step function [see S3 Fig(f)].

The above results suggest that our model of bacterial vesicle formation is robust with respect to the particular choice of $w(R)$, provided that $w(R)$ strongly suppresses the (relative) contribution of untethered membrane regions with small R to Eq. (1) in the main text.
