# Supplementary material for: The density of Braun’s Lipoprotein determines vesicle production in E. coli
Source: PLoS One. 2025 Sep 19;20(9):e0332156. doi: 10.1371/journal.pone.0332156 (PMC12448975; doi:10.1371/journal.pone.0332156)
Supplement: S2 Text — (PDF) [file pone.0332156.s002.pdf]

S2 Text. Fold change in steady state Lpp or GFP with protein degradation or mRNA degradation

We expect the number of Lpp proteins, L, in a given cell to be influenced primarily by the number of *lpp* mRNA, M_{lpp}, and the degradation rate of Lpp proteins, γ_{Lpp}, with translation of *lpp* mRNA into Lpp proteins occurring with some efficiency, ϵ_{lpp}. The number of Lpp proteins in a cell is thus expected to obey the equation

$$\frac{dL}{dt} = \epsilon_{lpp} M_{lpp} - \gamma_{\mathrm{Lpp}} L \, . \tag{S4}$$

Similarly, we expect the number of GFP proteins, G, to depend on the number of *gfp* mRNA, M_{gfp}, on the GFP degradation rate, γ_{GFP}, and on the mRNA translation efficiency, ϵ_{gfp}. We thus expect

$$\frac{dG}{dt} = \epsilon_{gfp} M_{gfp} - \gamma_{\mathrm{GFP}} G \, . \tag{S5}$$

At steady state, the number of Lpp, L_s, and GFP, G_s, are thus given by

$$L_s = \frac{\epsilon_{lpp} M_{lpp}}{\gamma_{\mathrm{Lpp}}} \tag{S6}$$

and

$$G_s = \frac{\epsilon_{gfp} M_{gfp}}{\gamma_{\mathrm{GFP}}} \, . \tag{S7}$$

In the main text, we are primarily concerned with estimating the fold change in the steady state levels of Lpp or GFP for a given condition, such as the level of induction, with respect to some reference state, such as WT protein levels. We can express these fold changes as

$$\frac{L_s^{cond}}{L_s^{ref}} = \frac{\frac{\epsilon_{lpp} M_{lpp}^{cond}}{\gamma_{\mathrm{Lpp}}}}{\frac{\epsilon_{lpp} M_{lpp}^{ref}}{\gamma_{\mathrm{Lpp}}}} = \frac{M_{lpp}^{cond}}{M_{lpp}^{ref}} \, , \tag{S8}$$

and

$$\frac{G_s^{cond}}{G_s^{ref}} = \frac{\frac{\epsilon_{gfp} M_{gfp}^{cond}}{\gamma_{\mathrm{GFP}}}}{\frac{\epsilon_{gfp} M_{gfp}^{ref}}{\gamma_{\mathrm{GFP}}}} = \frac{M_{gfp}^{cond}}{M_{gfp}^{ref}} \, . \tag{S9}$$

Note that factors of ϵ_{lpp}, ϵ_{gfp}, γ_{Lpp} and γ_{GFP} cancel out, with the fold change in the steady state protein number being equal to the fold change in the steady state mRNA number.

It is possible that the mRNA for Lpp and GFP degrade, potentially at different rates, which could lead to different steady state protein numbers. In this scenario, mRNA is produced at some promoter- and induction-level-dependent rate R, and degrades at some rate δ. The overall mRNA number, M, is then expected to obey

$$\frac{dM}{dt} = R - \delta M\,. \tag{S10}$$

In the steady state we thus have $M_s = \frac{R}{\delta}$, which allows us to calculate the fold change in the steady state mRNA number for some condition and reference point,

$$\frac{M_s^{cond}}{M_s^{ref}} = \frac{\frac{R^{cond}}{\delta}}{\frac{R^{ref}}{\delta}} = \frac{R^{cond}}{R^{ref}}\,. \tag{S11}$$

The above equation indicates that the fold change in the steady state mRNA number depends only on the fold change in R. In the main text, we were able to control R through the use of an inducible promoter, in our case the Tet repressor, which allowed us to control mRNA production by adding the inducer, aTc.

The above results suggest that measurements of relative protein number are equivalent to measurements of relative mRNA number, which are in turn equivalent to measurements of relative rates of mRNA production. In the main text, bacterial strains with inducible production of Lpp or GFP used the same promoter, and we therefore expect that the fold change in mRNA production will be the same for both strains under the same conditions (i.e., the same inducer concentration) and compared to the same reference state. This reasoning, combined with a single set of qPCR measurements of the *lpp* mRNA number compared to WT, allows us to estimate the fold change in Lpp number compared to WT conditions, at all induction levels measured in the GFP strain. In particular, combining Eqs. (S8), (S9), and (S11) we arrive at

$$\frac{L_s^{cond}}{L_s^{ref}} = \frac{G_s^{cond}}{G_s^{ref}}\,. \tag{S12}$$
