# Supplementary material for: The density of Braun’s Lipoprotein determines vesicle production in E. coli
Source: PLoS One. 2025 Sep 19;20(9):e0332156. doi: 10.1371/journal.pone.0332156 (PMC12448975; doi:10.1371/journal.pone.0332156)
Supplement: S3 Text — (PDF) [file pone.0332156.s003.pdf]

S3 Text. Variation of the critical radius with Lpp density

In the basic model of bacterial vesicle formation described in the main text, we considered a fixed value of R_c independent of the Lpp density ρ. The comparison between our theoretical predictions and experimental results in Fig. 4 shows that a constant R_c successfully predicts the observed fold changes in *E. coli* vesicle production at intermediate Lpp densities, and suggests that R_c may vary at low Lpp densities. Since we predict and measure vesicle production relative to WT *E. coli*, suitable changes in R_c cannot simply be read off from plots of vesicle production versus ρ as in Fig. 4, where each curve was calculated under the assumption of a constant R_c. In this section, we first explore how R_c would change if R_c was treated as a parameter to be fit to experimental data on vesicle production. Based on intuition gleaned from such fits, we then formulate a physical model of the variation of R_c with Lpp density. We show that this model yields good agreement with experiments for all measured ρ in Fig. 4.

In S7 Fig(a) we show the result of fitting the critical radius for vesicle formation to the experimental data in Fig. 4 at each Lpp density $\rho < 1$. The indicated, fitted values of R_c yield, within numerical accuracy, perfect agreement with the corresponding fold changes in vesicle production measured in our experiments in Fig. 4. At $\rho = 1$, we thereby fixed the critical radius to take the value $R_c = 12$ nm, which was shown in Fig. 4 to yield accurate predictions for bacterial vesicle production at intermediate Lpp expression levels. Not too surprisingly, we thus find in S7 Fig(a) that the fitted values of R_c are close to $R_c = 12$ nm at intermediate ρ. At small enough Lpp densities, however, we see deviations from $R_c = 12$ nm, with the fitted $R_c \approx 11$ nm at $\rho \approx 0.05$ and $R_c \approx 8.5$ nm at $\rho = 0$. Thus, contrary to what one might expect based on Fig. 4, the rapid increase in vesicle production at small ρ in Fig. 4 appears to be associated with a decrease in R_c relative to the value of R_c in WT *E. coli*.

What could give rise to the changes in R_c in S7 Fig(a)? According to the physical model of R_c in Eq. (2) of the main text, a decrease in the critical radius implies a decrease in the outer membrane bending rigidity, κ, and/or an increase in the turgor pressure across the outer membrane, P. In S7 Fig(b) we examine the variation in the normalized bending rigidity, $\bar{\kappa}$, and the normalized pressure, $\bar{P}$, with Lpp density, assuming one or the other to be solely responsible for the changes in critical radius suggested by our experiments. We define $\bar{\kappa}$ and $\bar{P}$ based on Eq. (2) via $\bar{\kappa}(\rho) = [R_c(\rho)/R_c(\rho = 1)]^3$ and $\bar{P} = [R_c(\rho = 1)/R_c(\rho)]^3$ with constant P and κ, respectively. From S7 Fig(b) we see that the decrease in critical radius suggested by our experiments would require a three-fold increase in P or, equivalently, a three-fold decrease in κ as the density of Lpp is decreased from $\rho = 1$ to $\rho = 0$. Considering that decreases in ρ tend to be associated with alterations in cell shape [see S5 Fig], changing the expression level of Lpp may have some effect on the pressure difference across the outer membrane. However, it is unclear how large such Lpp-induced changes in turgor pressure might be, or even whether a decrease in Lpp density produces an increase or a decrease in P. In contrast, it is known that a decrease in transmembrane protein density can yield a substantial decrease in membrane bending rigidity[1]. Considering how abundant Lpp is in WT cells, with most Lpp occurring in a transmembrane conformation[2], we therefore focus on a decrease in κ as the primary mechanism responsible for the decrease in R_c suggested by our data. We note, however, that changes in Lpp density may, in principle, affect both κ and P. We further explore changes in P with ρ in S8 Fig.

The upper panel in S7 Fig(b) indicates that the dependence of R_c on ρ implied by our experiments can be explained by a rapid decrease in κ as $\rho \to 0$, with an approximately constant κ at large enough values of ρ. In particular, we hypothesize that $\kappa(\rho)$ can be expressed in the form

$$\kappa(\rho) = \kappa_0 + A\,(1 - e^{-B\,\rho})\,, \tag{S13}$$

where the values of κ_0 and A follow from our experimental measurements of bacterial vesicle production at $\rho = 0$ and $\rho = 1$, and B is a fitting parameter. Note that we have $\kappa(0) = \kappa_0$ and $\kappa(1) = \kappa_0 + A\,(1 - e^{-B})$ at zero and WT Lpp densities, respectively. As suggested by our data on bacterial vesicle production, Eq. (S13) yields, for large enough values of B, a rapid decrease in κ as $\rho \to 0$ [see the inset in S7 Fig(c)]. Using Eq. (S13), we can express the critical radius in Eq. (2) in the simple analytic form

$$R_c(\rho) \approx 2\left[\frac{\kappa_0 + A\,(1 - e^{-B\,\rho})}{P}\right]^{\frac{1}{3}}. \tag{S14}$$

Defining the WT critical radius $R_c^{\mathrm{WT}} \equiv R_c(1)$ and the critical radius associated with $\rho = 0$, $R_c^{\Delta lpp} \equiv R_c(0)$, we rearrange Eq. (S14) to solve for κ_0 and A, resulting in

$$R_c(\rho) \approx \left[\left(R_c^{\Delta lpp}\right)^3\left(1 - \tilde{B}\right) + (R_c^{\mathrm{WT}})^3\,\tilde{B}\right]^{\frac{1}{3}}, \tag{S15}$$

where we have defined

$$\tilde{B} \equiv \frac{1 - e^{-B\,\rho}}{1 - e^{-B}}\,. \tag{S16}$$

S6 Fig(a) implies that $R_c^{\Delta lpp} \approx 8.5$ nm for $R_c^{\mathrm{WT}} = 12$ nm. In S7 Fig(c) we plot the resulting $R_c(\rho)$ in Eq. (S15) for several choices of B and, hence, $\tilde{B}$. Fitting Eq. (S15) to our data on bacterial vesicle production in Fig. 4 we find $B \approx 24$ (see Fitting routine). With this value of B, our model yields fold changes in vesicle production that closely match our experimental data for the entire interval $0 \le \rho \le 1$ [see S7 Fig(d)]. Similarly good agreement between theoretical and experimental results is obtained for $14 \lesssim B \lesssim 34$ (shaded region in S7 Fig(d)). Thus, our data on bacterial vesicle production suggests that Lpp helps to maintain outer membrane rigidity. In Eqs. (S8)–(S10) we derive an expression analogous to Eq. (S15) for a hypothetical scenario in which κ is kept fixed but P is allowed to vary with ρ, which can also yield good agreement with our experimental data, [see S8 Fig].

Previously, we hypothesized that the dependence of the critical radius R_c on the Lpp density ρ suggested by our experiments could be explained by a rapid decrease in κ with ρ as $\rho \to 0$, with an approximately constant κ at large enough values of ρ. Here, we consider an alternative scenario, in which we allow for a rapid increase in the turgor pressure P across the outer membrane as $\rho \to 0$, with an approximately constant P at large enough values of ρ. For simplicity, we thereby keep the value of κ fixed. Assuming, in analogy to $\kappa(\rho)$, that $P(\rho) = P_0 - C\,(1 - e^{-D\,\rho})$ [see the inset in S8 Fig(a)], the critical radius is given by

$$R_c(\rho) \approx 2\left(\frac{\kappa}{P_0 - C\,(1 - e^{-D\,\rho})}\right)^{\frac{1}{3}}, \tag{S17}$$

where P_0 and C are fixed by the values of $R_c^{\Delta lpp}$ and R_c^{WT} at $\rho = 0$ and $\rho = 1$ in S7 Fig(a), respectively, and D is a fitting parameter. Here, we have

$$R_c(\rho) \approx \left(\frac{1 - \widetilde{D}}{\left(R_c^{\Delta lpp}\right)^3} + \frac{\widetilde{D}}{(R_c^{\mathrm{WT}})^3} \right)^{-\frac{1}{3}}, \quad \text{(S18)}$$

where

$$\widetilde{D} \equiv \frac{1 - e^{-D\,\rho}}{1 - e^{-D}}\,. \quad \text{(S19)}$$

In analogy to S7 Fig(c), we plot in S8 Fig(a) $R_c(\rho)$ in Eq. (S18) for several choices of D and, hence, $\widetilde{D}$. The parameter D in Eq. (S18) is seen to control how rapidly $R_c(\rho)$ decreases with ρ as $\rho \to 0$. Fitting, as described previously, Eq. (S18) to our data on bacterial vesicle production in Fig. 4 of the main text, we find $D \approx 39$. With this value of D, our model yields fold changes in bacterial vesicle production that closely match our experimental data for the entire interval $0 \leq \rho \leq 1$ [see S8 Fig(b)]. Similarly good agreement between theoretical and experimental results is obtained for $29 \lesssim D \lesssim 49$ [shaded region in S8 Fig(b)]. Thus, we find good agreement with our experimental data not only if P is kept fixed and κ is allowed to vary with ρ, but also if κ is kept fixed and P is allowed to vary with ρ. However, as we have already noted, it is unclear how reducing the Lpp density would result in a sharp increase in turgor pressure across a narrow range of Lpp densities.

Fitting routine

At each Lpp density considered in this supplemental text, a critical radius $R_c(\rho)$ was applied to R-distributions of untethered membrane regions to calculate the fold change in bacterial vesicle number with respect to WT *E. coli*. In particular, the model parameter B in Eq. (S15) with Eq. (S16) was fit to the mean of the experimental replicates. The optimal value of B was determined using a weighted least squares fit to the experimentally measured fold changes in bacterial vesicle production, using the *lsqnonlin* fitting function (employing a trust region reflective algorithm) in the Optimization Toolbox (version 9.3) in MATLAB (version R2022a). The weight of a given data point was set to the inverse of the experimentally obtained standard error at that data point.

References

1. Lopez Mora N, Findlay HE, Brooks NJ, Purushothaman S, Ces O, Booth PJ. The membrane transporter lactose permease increases lipid bilayer bending rigidity. Biophys J. 2021;120: 3787–3794. doi:10.1016/j.bpj.2021.06.038

2. Cowles CE, Li Y, Semmelhack MF, Cristea IM, Silhavy TJ. The free and bound forms of Lpp occupy distinct subcellular locations in Escherichia coli. Mol Microbiol. 2011;79: 1168–1181. doi:10.1111/j.1365-2958.2011.07539.x
