# Supplementary material for: The density of Braun’s Lipoprotein determines vesicle production in E. coli
Source: PLoS One. 2025 Sep 19;20(9):e0332156. doi: 10.1371/journal.pone.0332156 (PMC12448975; doi:10.1371/journal.pone.0332156)
Supplement: S1 Fig — (PDF) [file pone.0332156.s004.pdf]

S1 Figure. Model predictions with modified crosslink numbers

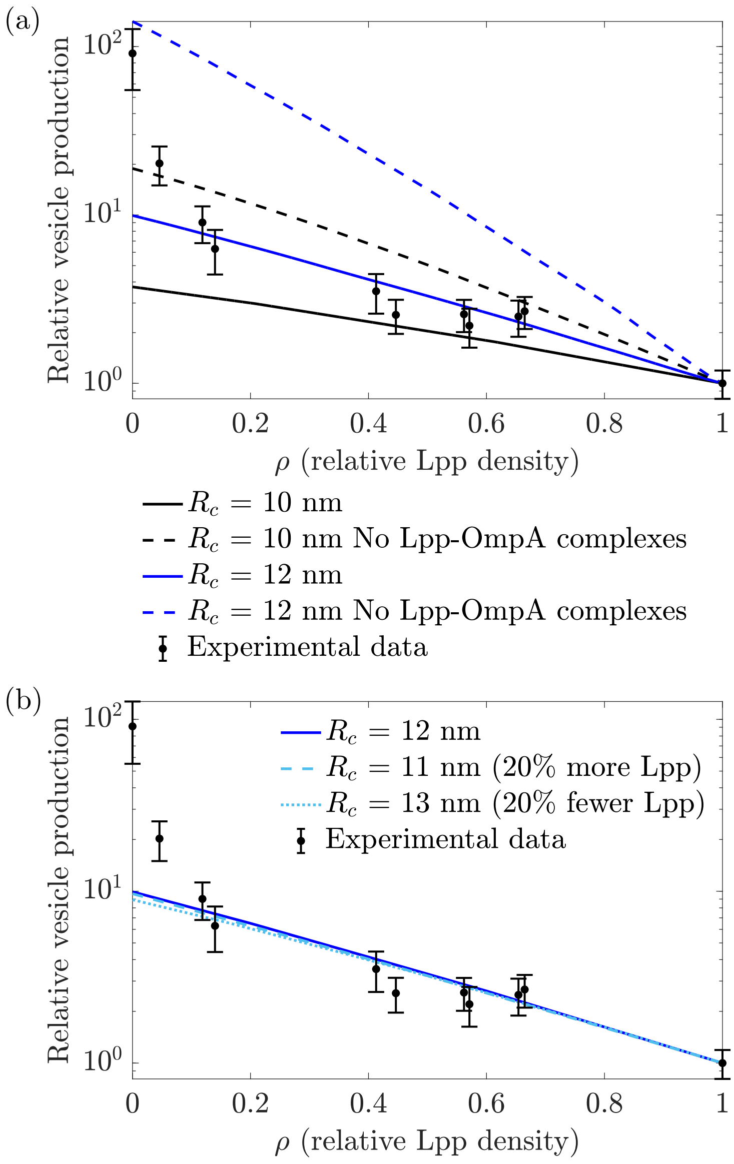

S1 Figure: Model predictions as in Figs. 2(d) and 4 of the main text, but without formation of Lpp-OmpA complexes, or with a 20% increase or decrease of Lpp crosslink number in WT *E. coli*. (a) Under the assumption that Lpp and OmpA do not form joint complexes, OmpA and Lpp all form independent crosslinks, which increases the total crosslink number at all Lpp densities (dashed curves). The solid curves show the corresponding results obtained with formation of Lpp-OmpA complexes and are identical to the curves for $R_c = 10$ nm and $R_c = 12$ nm in Figs. 2(d) and 4 of the main text. (b) Model predictions with a 20% increase (dashed curve) or a 20% decrease (dotted curve) in Lpp crosslinks in WT cells. The data points in (a) and (b) show our experimental results of the dependence of *E. coli* vesicle production on Lpp density, and are reproduced here from Fig. 4 of the main text for ease of comparison. Within our model of bacterial vesicle production, an increase in the overall crosslink number can effectively be absorbed into a decrease in the critical radius R_c. Conversely, a decrease in the overall crosslink number can effectively be absorbed into an increase in R_c.
