# Supplementary material for: The density of Braun’s Lipoprotein determines vesicle production in E. coli
Source: PLoS One. 2025 Sep 19;20(9):e0332156. doi: 10.1371/journal.pone.0332156 (PMC12448975; doi:10.1371/journal.pone.0332156)
Supplement: S2 Fig — (PDF) [file pone.0332156.s005.pdf]

S2 Figure. Model predictions under attractive or repulsive crosslink interactions

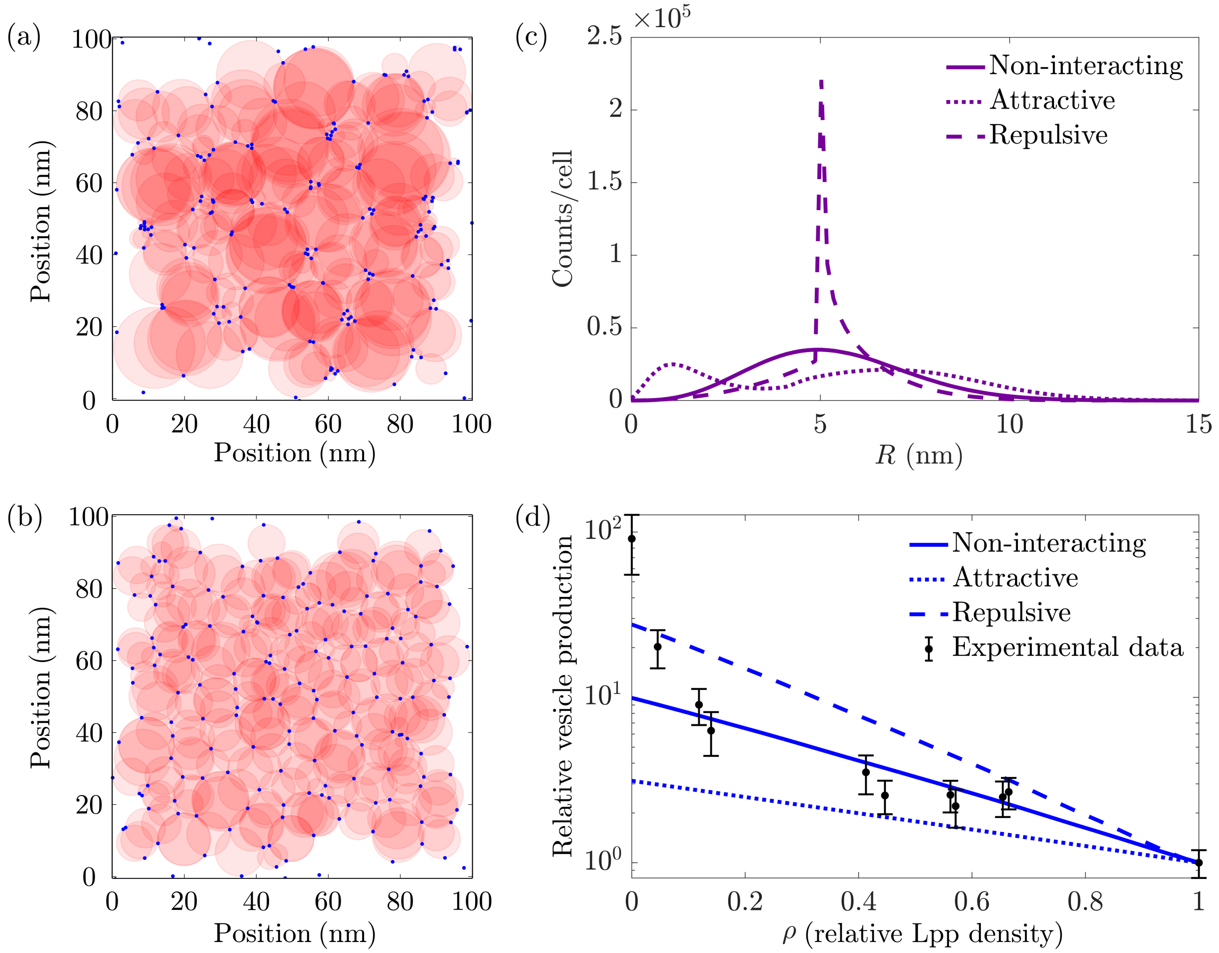

S2 Figure: Model results under the assumption that crosslinks attract each other and hence form clusters, or repel each other and hence more uniformly fill space. (a) Illustration of a simple model of crosslink attraction: As crosslinks are placed randomly within the membrane patch under consideration, any new crosslinks within 10 nm of an existing crosslink are redistributed radially about the closest existing crosslink using a Gaussian distribution with $4\,\sigma = 10$ nm, centered about this crosslink. For the case illustrated here we set $\rho = 1$. (b) Illustration of a simple model of crosslink repulsion: As crosslinks are placed randomly within the membrane patch under consideration, any new crosslinks within 10 nm of an existing crosslink are moved 10 nm radially outward from the closest existing crosslink. For the case illustrated here we set $\rho = 1$. (c) R-distributions of untethered outer membrane regions for $\rho = 1$ with attractive (dotted curve) and repulsive (dashed curve) crosslink interactions as in panels (a) and (b), respectively, and without any crosslink interactions as in the main text (solid curve). (d) Predicted fold changes in bacterial vesicle number with respect to WT *E. coli* as a function of Lpp density for attractive, repulsive, and noninteracting crosslinks assuming $R_c = 12$ nm, and corresponding results obtained from our experiments on bacterial vesicle production. We find that attractive or repulsive interactions between crosslinks tend to increase or decrease the critical radius implied by our model of bacterial vesicle production, respectively. The model results for noninteracting crosslinks and our experimental data on bacterial vesicle production are reproduced from Figs. 2 and 4 of the main text and included here for ease of comparison.
