# Supplementary material for: The density of Braun’s Lipoprotein determines vesicle production in E. coli
Source: PLoS One. 2025 Sep 19;20(9):e0332156. doi: 10.1371/journal.pone.0332156 (PMC12448975; doi:10.1371/journal.pone.0332156)
Supplement: S3 Fig — (PDF) [file pone.0332156.s006.pdf]

S3 Figure. Model predictions with alternative propensity functions

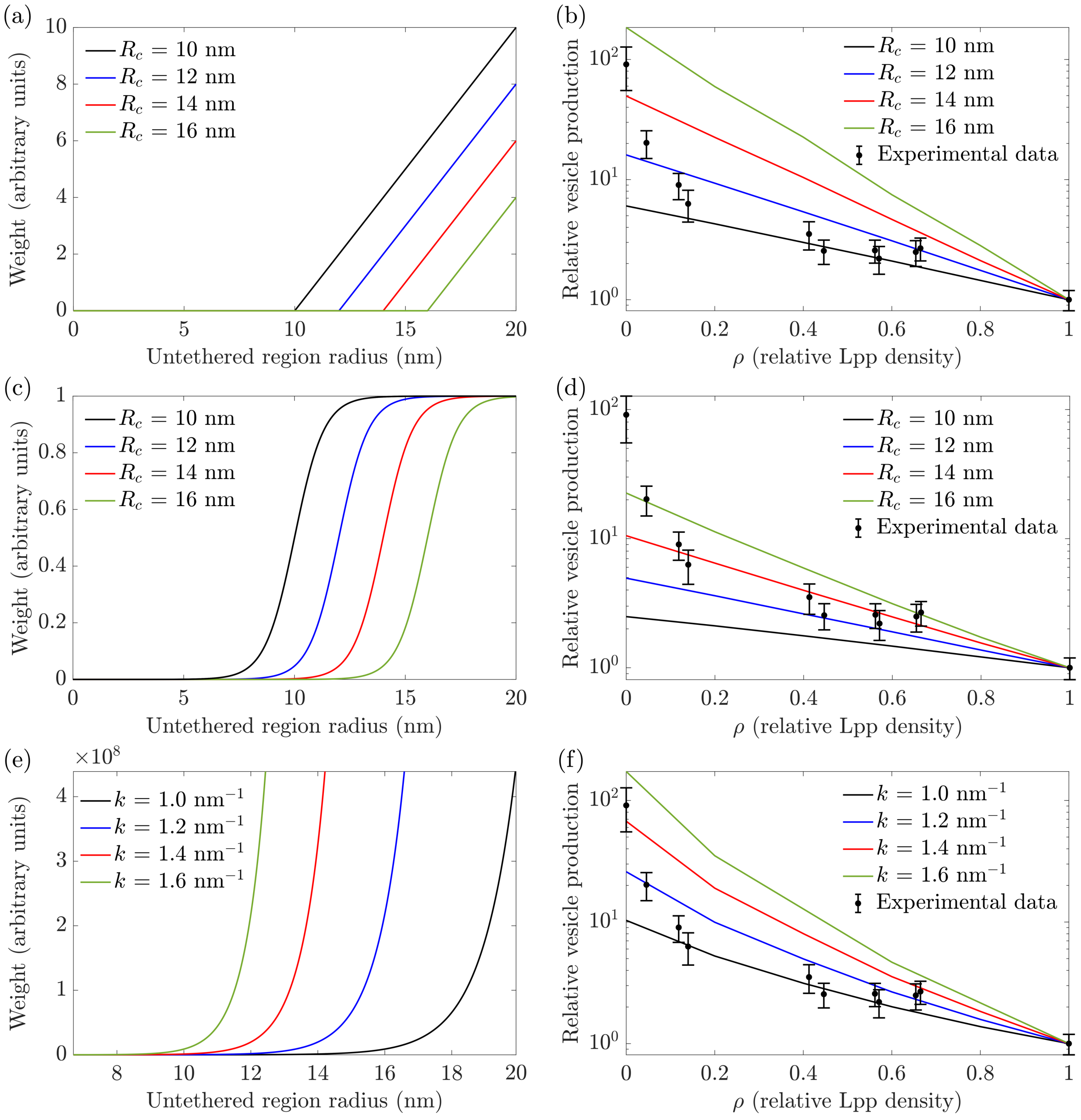

S3 Figure: Different choices for the propensity function $w(R)$ and corresponding fold changes in Eq. (1) of the main text. (a) Linear propensity function in Eq. (S1) for several choices of R_c. (b) Predicted fold changes in vesicle number with respect to WT *E. coli* as a function of Lpp density for the linear propensity functions in panel (a). (c) Sigmoidal propensity function in Eq. (S2) for several choices of R_c with $k = 1.5$. (d) Predicted fold changes in vesicle number with respect to WT *E. coli* as a function of Lpp density for the sigmoidal propensity functions in panel (c). (e) Exponential propensity function in Eq. (S3) for several choices of the scaling factor k. (f) Predicted fold changes in vesicle number with respect to WT *E. coli* as a function of Lpp density for the exponential propensity functions in panel (e).
