# Supplementary material for: The density of Braun’s Lipoprotein determines vesicle production in E. coli
Source: PLoS One. 2025 Sep 19;20(9):e0332156. doi: 10.1371/journal.pone.0332156 (PMC12448975; doi:10.1371/journal.pone.0332156)
Supplement: S4 Fig — (PDF) [file pone.0332156.s007.pdf]

S4 Figure. Fluorescence curves and relative *lpp* expression

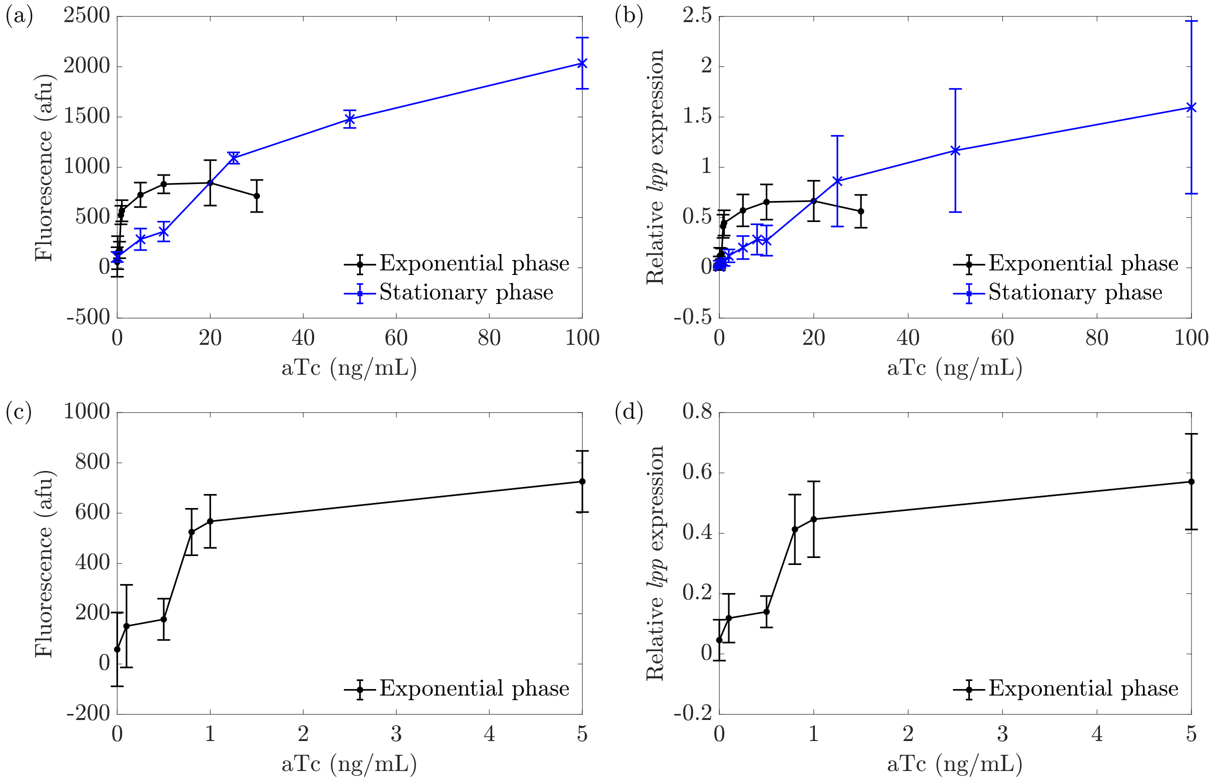

S4 Figure: GFP fluorescence as a function of the concentration of the inducer used here, aTc, and corresponding relative *lpp* expression level determined using a combination of GFP and qPCR measurements, as described in the main text. (a) GFP fluorescence with background subtracted and fluorescence normalized to OD_{600} for both exponential phase and stationary phase cells. (b) Estimates of relative *lpp* expression, based on the GFP fluorescence measurements in panel (a) and qPCR measurement of *lpp* expression at 5 ng/mL aTc, as described in the main text. (c) Exponential phase data from panel (a), with a limited aTc-range so as to better show results near 0 ng/mL aTc. (d) Exponential phase data from panel (b) with, as in panel (c), a limited aTc-range. The error bars in panels (a) and (c) denote standard errors, $n \geq 3$. The error bars in panels (b) and (d) denote propagated standard errors from the qPCR measurement, $n = 4$, and the GFP fluorescence measurements in panel (a), $n \geq 3$.
