# Supplementary material for: The density of Braun’s Lipoprotein determines vesicle production in E. coli
Source: PLoS One. 2025 Sep 19;20(9):e0332156. doi: 10.1371/journal.pone.0332156 (PMC12448975; doi:10.1371/journal.pone.0332156)
Supplement: S5 Fig — (PDF) [file pone.0332156.s008.pdf]

S5 Figure. *E. coli* aspect ratio is restored to WT values at high enough levels of plasmid induction

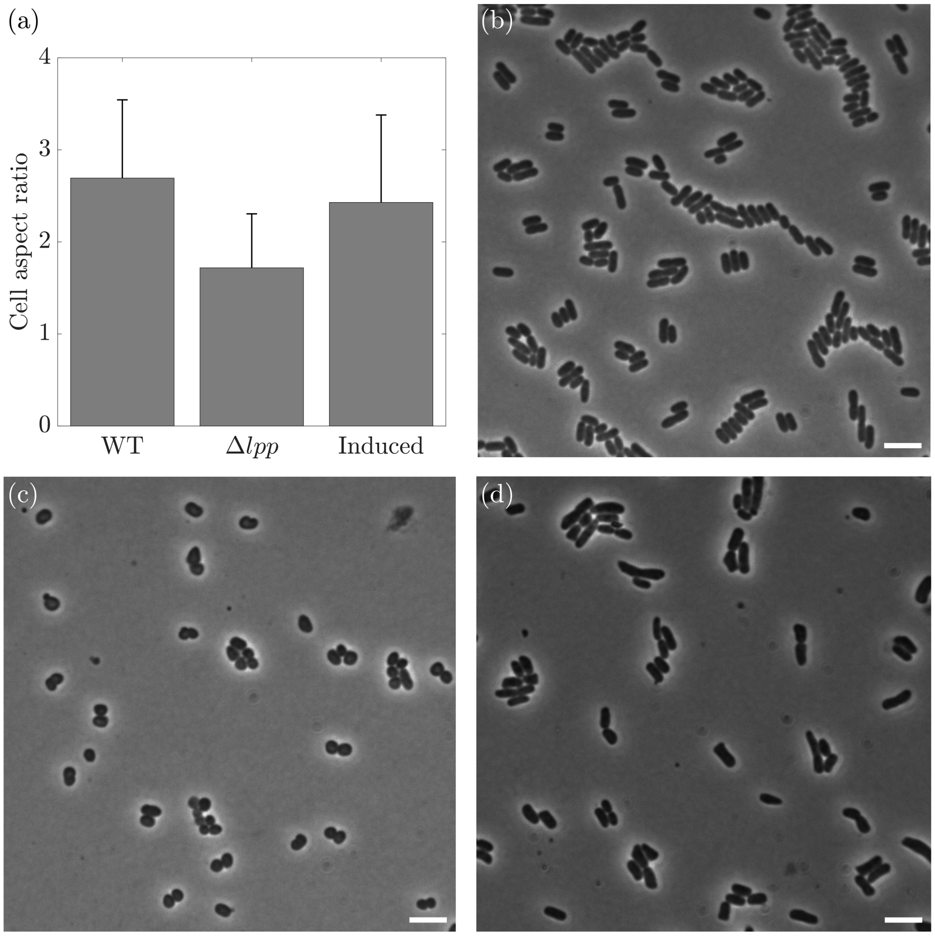

S5 Figure: The *E. coli* cellular aspect ratio is restored to WT values at high enough levels of plasmid induction, indicating that the plasmid system used here produces functional Lpp. (a) Aspect ratio of WT, *Δlpp*, and plasmid-containing cells (see main text for details) induced with 100 ng/mL aTc. Induced cells have an aspect ratio comparable to that of WT cells. Aspect ratios were determined using Weka segmentation and shape analysis in Fiji. Error bars represent standard deviations; $n \geq 958$. (b) Phase contrast image of the WT cells used in this study, *E. coli* MG1655. (c) Phase contrast image of *Δlpp E. coli* demonstrating the more rounded shape typical of *E. coli* strains deficient in outer membrane crosslinks. (d) Phase contrast image of plasmid-containing cells induced with 100 ng/mL aTc (~70% of WT value), demonstrating rod-shaped cells similar in appearance to the WT strain in panel (b). Scale bars, 5 μm.
