# Supplementary material for: The density of Braun’s Lipoprotein determines vesicle production in E. coli
Source: PLoS One. 2025 Sep 19;20(9):e0332156. doi: 10.1371/journal.pone.0332156 (PMC12448975; doi:10.1371/journal.pone.0332156)
Supplement: S6 Fig — (PDF) [file pone.0332156.s009.pdf]

S6 Figure. Stationary phase measurements of vesicle production

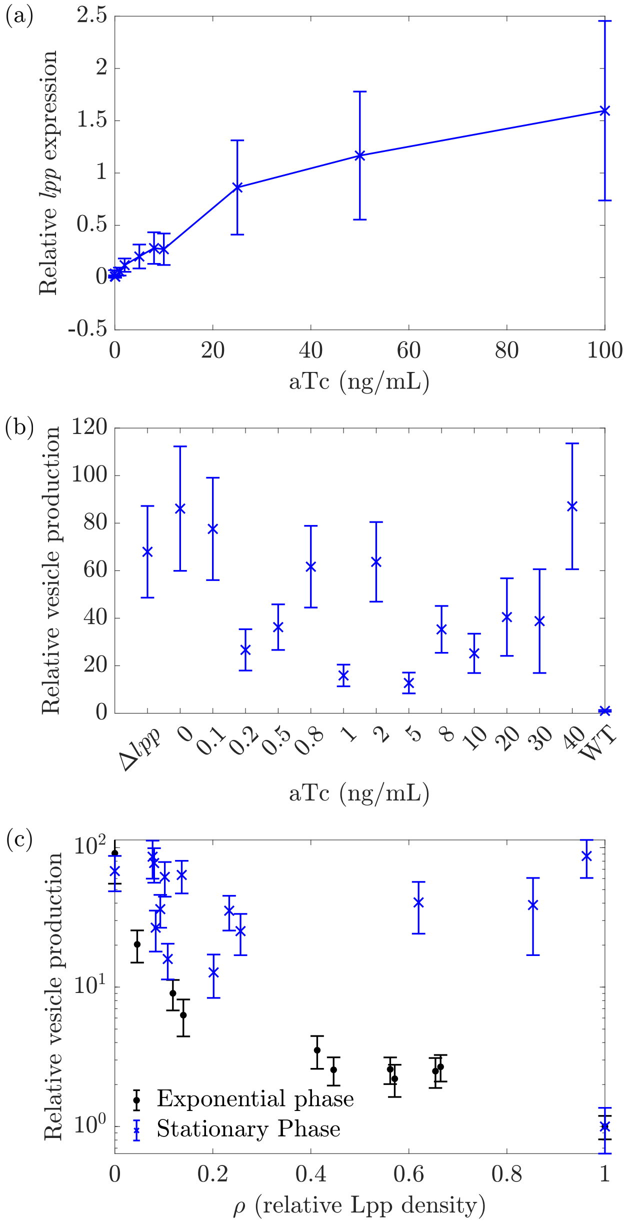

S6 Figure: Stationary phase measurements of the fold change in bacterial vesicle number with respect to WT *E. coli* as a function of Lpp density. To harvest vesicles from *E. coli* in stationary phase, overnight cultures were diluted 1:100 in fresh media and grown for approximately 6 hours. Subsequently, cultures were diluted 1:100 into fresh media, aTc was added for experiments that required induction of *lpp* expression, and cells were grown overnight a second time. (a) *lpp* expression levels relative to WT as a function of aTc concentration, measured as in the main text via fluorescent reporters and qPCR. High concentrations of the inducer aTc restore WT or greater levels of *lpp* expression. Lower concentrations give intermediate levels of *lpp* expression. (b) Fold change in bacterial vesicle number with respect to WT *E. coli* over a range of aTc concentrations. No aTc concentrations tested restored WT levels of vesicle production. (c) Experimental measurements of fold changes in bacterial vesicle number with respect to WT *E. coli* as a function of Lpp density in both stationary phase and exponential phase. Stationary phase measurements show no discernable trend, and fail to restore WT levels of vesicle production. Error bars represent standard error; $n \geq 3$.
