# Supplementary material for: The density of Braun’s Lipoprotein determines vesicle production in E. coli
Source: PLoS One. 2025 Sep 19;20(9):e0332156. doi: 10.1371/journal.pone.0332156 (PMC12448975; doi:10.1371/journal.pone.0332156)
Supplement: S7 Fig — (PDF) [file pone.0332156.s010.pdf]

S7 Figure. Variation of the critical radius with Lpp density due to varying membrane bending rigidity

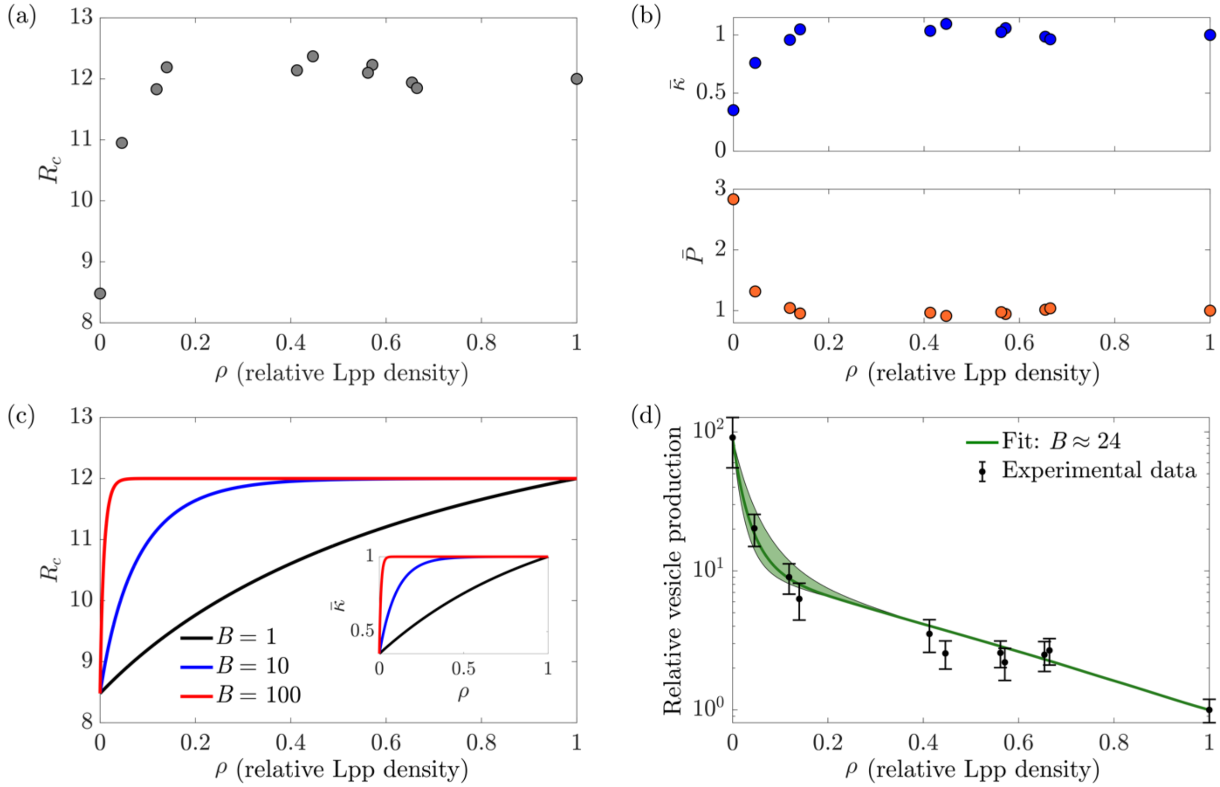

S7 Figure: Effect of changes in the critical radius with Lpp density. (a) Fits of the critical radius for vesicle formation to the data in Fig. 4 at each Lpp density $\rho < 1$ measured in experiments, assuming $R_c = 12$ nm at $\rho = 1$. (b) Normalized bending rigidity (top panel) and normalized pressure (bottom panel) implied by Eq. (S4) and panel (a) with P and κ held fixed, respectively. (c) Critical radius as a function of Lpp density in Eq. (S3) with Eq. (S4) for the indicated values of the fitting parameter B. (inset) Normalized bending rigidity as a function of Lpp density for the values of B indicated in the main panel. (d) Fold change in bacterial vesicle number with respect to WT *E. coli* as a function of Lpp density calculated from Eq. (S3) with Eq. (S4) using $B \approx 24$, and corresponding experimental data reproduced from Fig. 4. The shaded region indicates our model results for $B \approx 14$ to $B \approx 34$. Error bars represent standard error; $n \geq 3$.
