# Supplementary material for: The density of Braun’s Lipoprotein determines vesicle production in E. coli
Source: PLoS One. 2025 Sep 19;20(9):e0332156. doi: 10.1371/journal.pone.0332156 (PMC12448975; doi:10.1371/journal.pone.0332156)
Supplement: S8 Fig — (PDF) [file pone.0332156.s011.pdf]

S8 Figure. Variation of the critical radius with Lpp density due to varying turgor pressure

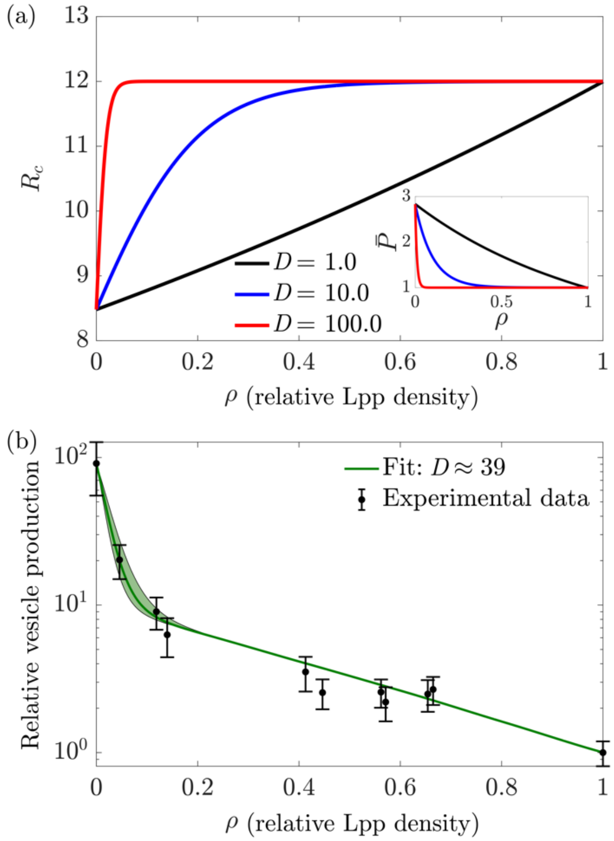

S8 Figure: Model results for an increasing turgor pressure across the outer membrane with decreasing Lpp density. (a) Critical radius for vesicle formation as a function of Lpp density resulting from Eq. (S9) with Eq. (S10) for the indicated choices of D. (inset) Normalized turgor pressure, $\bar{P} = [R_c(\rho = 1)/R_c(\rho)]^3$, as a function of Lpp density for the indicated choices of D. (b) Fold change in bacterial vesicle number with respect to WT *E. coli* as a function of Lpp density calculated using Eq. (S9) with Eq. (S10) and $D \approx 39$, and corresponding experimental data reproduced from Fig. 4 of the main text. The shaded region indicates our model results for $D \approx 29$ to $D \approx 49$. Error bars represent standard error; $n \geq 3$.
