# Supplementary material for: The density of Braun’s Lipoprotein determines vesicle production in E. coli
Source: PLoS One. 2025 Sep 19;20(9):e0332156. doi: 10.1371/journal.pone.0332156 (PMC12448975; doi:10.1371/journal.pone.0332156)
Supplement: S9 Fig — (PDF) [file pone.0332156.s012.pdf]

S9 Figure. Ultracentrifugation has a minimal effect on vesicle size and concentration

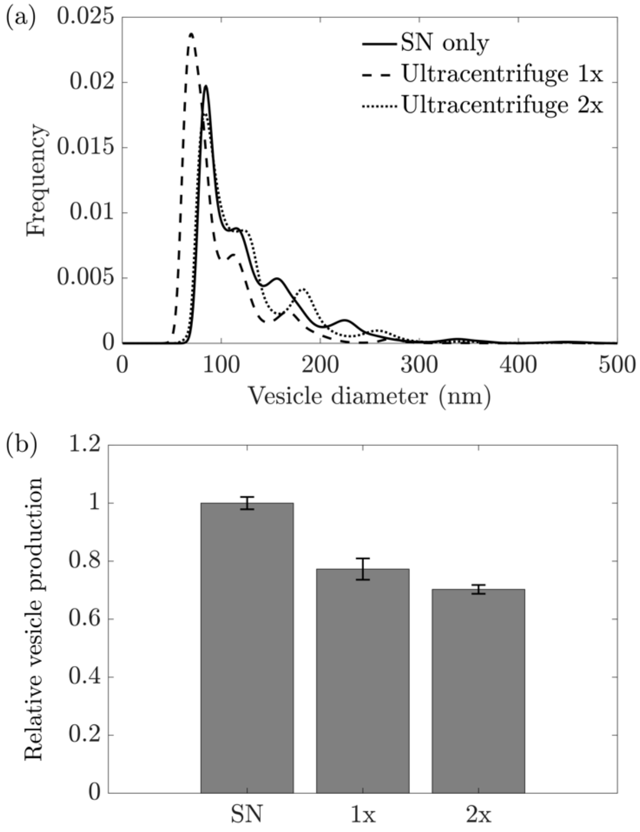

S9 Figure: Ultracentrifugation has a minimal effect on *E. coli* vesicle size and concentration as measured by nanoparticle tracking analysis. (a) Vesicle size distribution of *Δlpp* cells for culture supernatant (SN), after the SN was processed via one round of ultracentrifugation (1x), and after the SN was processed via two rounds of ultracentrifugation (2x). (b) Total vesicle concentrations for *Δlpp* cells relative to initial, SN vesicle concentration after one (1x) and two (2x) rounds of processing via ultracentrifugation. Note that, in prior studies that did not use nanoparticle tracking analysis to measure vesicle counts, ultracentrifugation was required to sufficiently concentrate vesicles for particle density measurements (main text Refs. [9,18]).
