# Supplementary material for: The density of Braun’s Lipoprotein determines vesicle production in E. coli
Source: PLoS One. 2025 Sep 19;20(9):e0332156. doi: 10.1371/journal.pone.0332156 (PMC12448975; doi:10.1371/journal.pone.0332156)
Supplement: S1 Table — (PDF) [file pone.0332156.s013.pdf]

S1 Table. qPCR primers used in this study.

Primers	Sequence
lpp-qPCR-F	CTGTCTTCTGACGTTCAGACTC
lpp-qPCR-R	ACGAGCTGCGTCATCTTTAG
idnT-qPCR-F	GTTGTATGTTGGCGTACCAATG
idnT-qPCR-R	GTTCCGAGATTAGCCTCAAAGA
