# Supplementary material for: The density of Braun’s Lipoprotein determines vesicle production in E. coli
Source: PLoS One. 2025 Sep 19;20(9):e0332156. doi: 10.1371/journal.pone.0332156 (PMC12448975; doi:10.1371/journal.pone.0332156)
Supplement: S2 Table — (PDF) [file pone.0332156.s014.pdf]

S2 Table. Plasmid primers used in this study.

Primers	Sequence
pDSG372-lpp-SQ-F	AGCTATCGAATTATTTGATCACCAAGGTG
pDSG372-lpp-SQ-R	TCGGTGAGTTTTCTCCTTCATTACAGAAAC
lpp-pDSG372-F	GAAATACTAGATGAAAGCTACTAAACTGGTACTGGGC
lpp-pDSG372-R	ACTAGTATTATTACTTGCGGTATTTAGTAGCCATGTTGTC
pSC101-pDSG372terminator-vector-F	ACCGAAAAGACCATTATTATCATGACATTAACCTATAAAAATAGGC
pDSG372terminator-pSC101-R	ATAATAATGGTCTTTTCGGTTTTAAAGAAAAAGGACAGGGTG
PSC101-tetR-empty-R	ACTAGTATTACTAGTATTTCTCCTCTTTCTCTAGTAGTGCTCAG
pDSG372-tetR-empty-F	GAAATACTAGTAATACTAGTAGCGGCCGCTGCAG
pSC101-pDSG372tetR-vector-R	TTTTGCACCATTTCTTAGACGTCAGGTGGCACTTTTC
pDSG372tetR-pSC101-F	GTCTAAGAAATGGTGCAAAACCTTTGCGGTATG
pSC101-Tet-F:	**GAGCTGTACAAGTAA**TAATACTAGTAGCGGCCGCTG
pSC101-Tet-R:	**GCCCTTGCTCACCAT**CTAGTATTTCTCCTCTTTCTCTAGTAGTG
GFP-F:	**GAGGAGAAATACTAG**ATGGTGAGCAAGGGCGAGGA
GFP-R:	**CCGCTACTAGTATTA**TTACTTGTACAGCTCGTCCATGC
